# *Silybum marianum* peptide-rich fraction alleviates acute liver injury by inhibiting TRAF3/NIK/NF-κB and is associated with changes in gut microbiota composition

**DOI:** 10.3389/fphar.2026.1915943

**Published:** 2026-10-05

**Authors:** Ananda, Wenyan Han, Khurelbaatar Nyamdavaa, Xian Li, Tsend-Ayush Damba, Xiulan Su

**Affiliations:** 1 International School of Mongolian Medicine, Mongolian National University of Medical Sciences, Ulaanbaatar, Mongolia; 2 Clinical Laboratory, The Second Affiliated Hospital, Inner Mongolia Medical University, Hohhot, China; 3 Inner Mongolia Bioactive Peptide Engineering Laboratory, Clinical Medical Research Center, The Affiliated Hospital of Inner Mongolia Medical University, Hohhot, China

**Keywords:** acute liver injury, bioactive peptipeptide, gut microbiota, hepatoprotection, *Silybum marianum*

## Abstract

Acute liver injury (ALI) is a severe and prevalent condition that can progress to irreversible liver damage and failure. This study aimed to investigate the prophylactic hepatoprotective potential of *Silybum marianum* peptides (SMP) in ALI and elucidate its underlying mechanisms. Using a carbon tetrachloride (CCl_4_)-induced ALI mouse model, we evaluated the dose-dependent effects of SMP. Additionally, we explored the modulatory effects of SMP on the NF-κB pathway, gut microbiota composition, and metabolite profiles. The role of SMP in the TRAF3/NIK/NF-κB signaling axis was further validated in a NIK-overexpressing Hepa1-6 cell model. Our results demonstrated that 14-day SMP administration did not significantly affect body weight, fasting blood glucose levels, or liver and spleen weights, and no significant difference in serum ALT activity was observed. Notably, SMP (200 mg/kg) significantly alleviated CCl_4_-induced liver injury, as evidenced by enhanced hepatic antioxidant capacity, reduced expression of proinflammatory cytokines, and attenuated immune cell infiltration. Furthermore, SMP was associated with changes in gut microbiota composition and community structure in CCl_4_-induced ALI mice. Mechanistically, SMP suppressed the TRAF3/NIK/NF-κB pathway. These findings highlight SMP as a promising candidate for the prevention of CCl_4_-induced ALI, acting through multimodal mechanisms involving anti-inflammatory, antioxidant, and gut microbiota-associated pathways.

## Highlights

- *Silybum marianum* peptides alleviated acute liver injury by inhibiting the TRAF3/NIK/NF-κB signaling pathway.
- *Silybum marianum* peptides regulated the gut microbiota composition and metabolite profile in acute liver injury mice.

## Introduction

Acute liver injury (ALI) is a severe clinical condition that can progress to liver failure and is associated with high mortality. It is commonly triggered by viral infections, exposure to toxins, excessive alcohol intake, or drug abuse (Tujios et al., 2022; Chayanupatkul and Schiano, 2020). ALI is typically marked by hepatic inflammation, functional impairment, and metabolic dysregulation (Tujios et al., 2022). Current management often involves a comprehensive approach including prolonged rest, nutritional support, pharmacological interventions, and, in extreme cases, liver transplantation (Beech et al., 2023; Piccioni et al., 2020; Liu et al., 2021; Yoo et al., 2023). Despite these efforts, prevention options for ALI remain limited, and the underlying pathological mechanisms require further elucidation.

*Silybum marianum* (commonly known as milk thistle) has gained recognition as a medicinal plant with notable efficacy in both preventing and mitigating liver injury by intense oxidative stress such as inflammatory liver disease and cirrhosis (Palomino et al., 2017; Abenavoli et al., 2010). High-performance liquid chromatographic analysis shows flavonolignans silychristin and silibinin A and B as major components. Its active extract, silymarin, has demonstrated protective potential against various liver diseases, including cirrhosis, chronic hepatitis, and nonalcoholic fatty liver disease (NAFLD) (Jaffar et al., 2024; Navarro et al., 2019). Silibinin alone also recovered reduced glutathione and antioxidant enzymes (Palomino et al., 2017). Preliminary studies suggest that total extracts of *S. marianum*, silymarin and silibinin can inhibit hepatocarcinogenesis and suppress the growth of hepatocellular carcinoma by modulating key signaling pathways, such as the hepatocyte growth factor/c-Met (HGF/c-Met), Wnt/β-catenin, and phosphoinositide 3-kinase/protein kinase B/mammalian target of rapamycin (PI3K/Akt/mTOR) pathways (Yassin et al., 2022; Gültekin, 2023; Feng et al., 2016). Additionally, the protective effects of silibinin against ethanol- or acetaldehyde-induced damage in liver cell lines are linked to the suppression of mitochondrial fission (Song et al., 2022). Despite these promising findings, there remains a significant gap in research focused on the further development and evaluation of natural bioactive compounds derived from *S. marianum*.

Bioactive peptides are diverse in their biological activities and may exhibit favorable bioavailability and safety profiles, depending on their sequence and physicochemical properties. As fundamental components of biological systems, peptides play important roles in regulating physiological functions and metabolic processes. These protein-derived functional fragments offer superior bioavailability compared to free amino acids and possess diverse biological functions, including immune regulation and promotion of cellular proliferation. Emerging as a novel protective/therapeutic approach, bioactive peptides have demonstrated significant potential in mitigating liver injury. Current research has revealed that specific bioactive peptides can effectively suppress the nuclear factor kappaB (NF-κB) signaling pathway, thereby substantially reducing the expression of pro-inflammatory cytokines. Through this mechanism of inhibiting both the NF-κB pathway and its downstream inflammatory mediators, bioactive peptides exhibit promising protection prospects for the prevention of liver injury. Recently, bioactive peptides from various sources, including *Musculus senhousei*, *Litopenaeus vannamei*, and phycobiliproteins, have shown potential in alleviating liver damage and improving liver metabolism (Xiao et al., 2023; Moradian et al., 2022; Liu J et al., 2022).

*Silybum marianum (L.) Gaertn,* a herbaceous plant with a long history in traditional medicine for the treatment of hepatobiliary diseases, particularly in Europe, which has attracted attention for its remarkable protective effect (Fernández et al., 2021). *Silybum marianum* peptides (SMP), a novel bioactive peptide complex extracted from *S. marianum* for the first time by us, has the potential to cope with acute liver injury, and its mechanism of action warrants further investigation. The complete composition and sequences of *SMP* remain to be characterized.

Ubiquitination is a crucial posttranslational modification in which small ubiquitin (Ub) proteins are attached to target proteins, regulating their stability, activity, localization, and degradation (Song and Luo, 2019). This process involves the enzymes E1 (ubiquitin-activating enzyme), E2 (ubiquitin-conjugating enzyme), and E3 (ubiquitin ligase), with E3 ligases responsible for recognizing specific target proteins and linking ubiquitin to lysine residues on those proteins. Tumor necrosis factor receptor-associated factor 3 (TRAF3) is a key E3 ubiquitin ligase that regulates the NF-κB signaling pathway, influencing cellular immune responses, apoptosis, and inflammation; thus, TRAF3 has also become an important target for alleviating liver injury (Shiode et al., 2023; Zhou et al., 2021). NF-κB activation, which is triggered by various stimuli, such as oxidative stress and endotoxins, leads to the transcription of proinflammatory cytokines, exacerbating liver injury (Luedde and Schwabe, 2011). Additionally, NF-κB-inducing kinase (NIK), also known as MAP3K14, plays important roles in cell senescence, longevity extension, antioxidant stress, and energy metabolism regulation (Zhang et al., 2022). NIK has been found to activate the transcription of NF-κB and Nrf2, both of which play crucial roles in the development and recovery of liver injury (Zhang et al., 2022; Zhong et al., 2021). Recent studies have indicated that *S. marianum* can modulate inflammatory responses by influencing the NF-κB pathway in liver diseases (Feng et al., 2019). Furthermore, research on silymarin has demonstrated its capacity to alleviate liver inflammation induced by carbon tetrachloride (CCl_4_) through the modulation of the gene expression of key inflammatory cytokines, including tumor necrosis factor-alpha (TNF-α), interleukin-6 (IL-6), cyclooxygenase-2 (COX-2), and transforming growth factor-beta (TGF-β) (El-Kot et al., 2023). However, the specific role of SMP in regulating the TRAF3/NIK/NF-κB axis in ALI and the potential for protection targeting of this pathway by SMP remain largely unexplored, suggesting a promising avenue for further research into novel protection strategies for ALI.

Emerging evidence has highlighted the importance of the gut‒liver axis in regulating liver function and in the pathogenesis of liver diseases (Albillos et al., 2020; Hsu and Schnabl, 2023). The gut microbiota plays a crucial role in modulating immune responses, inflammation, and the metabolism of bile acids and other metabolites that influence liver health. Dysbiosis, or an imbalance in the gut microbiota, has been shown to exacerbate liver injury, including in models of acute liver damage (Chen et al., 2021). The gut microbiota can influence liver inflammation through the release of microbial metabolites, which can either promote or mitigate liver injury (Beaumont et al., 2018). Recent studies suggest that therapeutic interventions targeting the gut microbiota may have the potential to alleviate liver diseases (Parthasarathy et al., 2026). For example, silibinin has been shown to exert beneficial effects on serum lipids, bile acids, and the gut microbiota in mice fed a methionine-choline-deficient diet (Bai et al., 2024). However, the impact of SMP on the gut microbiota in the context of ALI remains largely unexplored, creating an opportunity for further investigation into novel prevention strategies for ALI.

This study aimed to evaluate the hepatoprotective effects of SMP in a CCl_4_-induced ALI model, with a focus on their ability to modulate the TRAF3/NIK/NF-κB pathway and regulate the gut microbiota. By targeting inflammation and the gut microbiota, this research could lead to the development of more effective and safer prevention for ALI, offering a promising alternative to current therapies.

## Materials and methods

### *Silybum marianum* and SMP

Xin Hong Biotechnology Co., Ltd., produces SMP. The preparation process is as follows: the dried *S. marianum* seeds (Xin Hong Biotechnology Co., Ltd., China) were cleaned to remove impurities and then pulverized using a high-speed grinder to pass through a 60-mesh sieve. An appropriate amount of the resulting powder was mixed with 10 times its volume of deionized water (e.g., 100 g powder with 1,000 mL water) and stirred thoroughly. Defatting was performed by adding petroleum ether at a 1:3 (w/v) ratio, followed by magnetic stirring at room temperature for 30 min; this process was repeated twice. The defatted powder was air-dried to remove residual solvent. The dried powder was then redispersed in deionized water, the pH adjusted to 8.0, and alcalase was added at 2% of the substrate weight (i.e., 2 g enzyme per 100 g powder). Enzymatic hydrolysis was carried out in a water bath shaker at 50 °C for 4 h. To terminate enzymatic activity, the mixture was heated at 95 °C for 10 min. After cooling, the hydrolysate was centrifuged at 8,000 rpm for 15 min, and the supernatant was collected. This supernatant was fractionated using an ultrafiltration system with 10 kDa and 3 kDa molecular weight cut-off membranes. The fraction below 3 kDa was collected as the target SMP segments. The peptide solution was concentrated to one-fourth of its original volume using a rotary evaporator and then freeze-dried to obtain a light yellow SMP powder, which was sealed and stored in a cool, dry, and dark environment for future use. Through high-performance liquid chromatography-tandem mass spectrometry (HPLC-MS/MS) analysis, 3 peptide chains were identified from silymarin with the following amino acid sequences: WFNRNADEEEGGEL, GPHYNPHGKEHGAPDDEVR and TILPDSEGAIDGHL, with identification scores of [85.3/2.1e-5], [112.7/3.4e-8], and [67.9/1.2e-4], respectively (FDR <1%).

### Construction of an animal model of ALI

All animal procedures were approved by the Ethics Committee of the Biomedical Research Ethics Committee of Inner Mongolia Medical University (YKD202405065), and were performed in compliance with the Guide for the Care and Use of Laboratory Animals. Adult male C57BL/6 J mice aged 8 weeks (weight: 20–25 g) were obtained from Shanghai SLAC Laboratory Animal Co., Ltd., (Shanghai, China), acclimatized for 7 days before experimentation, and exposed to 22 °C ± 2 °C indoor temperature and 50% relative humidity, and the light cycle was set to bright for 12 h and dark for 12 h. The mice were given free access to drinking water and standard maintenance chow. Mice were housed 5 animals per cage, and cages were randomly assigned to the experimental groups. The ALI model was established by intraperitoneal injection of 10 μL/g body weight of 10% CCl_4_ (v/v; 99.9%; Sigma-Aldrich, United States, Cat. No. 319961) prepared in highly refined, low-acidity olive oil (Sigma-Aldrich, United States, Cat. No. O1514). For toxicity assessment, mice were humanely euthanized on Day 13 for sample collection. For the remaining experiments, mice received CCl_4_ on Day 14 and were humanely euthanized 16 h after CCl_4_ administration for sample collection (Supplementary Figure S1). To ensure deep anesthesia and prevent any pain or distress, mice were first anesthetized via intraperitoneal injection with a mixture of ketamine (100 mg/kg) and xylazine (10 mg/kg). Following confirmation of deep anesthesia by the absence of pedal and corneal reflexes, euthanasia was performed by cervical dislocation. This method is consistent with the AVMA Guidelines for the Euthanasia of Animals. Subsequently, blood was collected via cardiac puncture, and liver tissues were rapidly excised. Tissue samples were either immediately frozen in liquid nitrogen and stored at −80 °C for molecular analysis or fixed in 10% neutral buffered formalin for histological examination. For microbiome analysis, cecal contents were collected from individual mice, with samples obtained from multiple cages to minimize potential cage effects. This study was carried out in compliance with the Animal Research: Reporting of *In Vivo* Experiments (ARRIVE) guidelines.

### Grouping methods

Mice were randomly assigned to the experimental groups using a random-number method. Investigators involved in animal treatment, sample collection, histological and immunofluorescence imaging, image quantification, and statistical analysis were blinded to group allocation. No animals or samples were excluded from the final analyses, and no attrition occurred during the study. The control group received a daily intragastric dose of normal saline from Days 1–14 and a single intraperitoneal dose of olive oil on Day 14. The CCl_4_ group was similarly administered normal saline daily, with an additional 10 μL/g body weight of 10% CCl_4_ (in olive oil) administered intraperitoneally on the final day. For the preventive SMP groups, mice received SMP at 50, 100, or 200 mg/kg by intragastric administration once daily for 14 consecutive days. On Day 14, following the final SMP administration, mice were challenged intraperitoneally with 10 μL/g body weight of 10% CCl_4_ in olive oil.

### Liver and spleen wet weights and indices and blood glucose monitoring

The livers and spleens of the mice were dissected, and the organs were washed gently with normal saline, wiped and weighed. Organ indices were calculated as follows:

$$\text{Liver organ index}=\text{liver weight }\left(g\right)/\text{body weight }\left(g\right).$$

$$\text{Spleen organ index}=\text{spleen weight }\left(g\right)/\text{body weight }\left(g\right).$$

The fasting blood glucose concentration of the mice was monitored daily using a portable blood glucose meter (Bayer AG, Leverkusen, Germany.). Approximately 5 µL of blood was collected from the tail tip of mice each time.

### H & E staining

The liver and spleen tissues fixed with neutral 10% formalin were subjected to ethanol gradient dehydration, transparency, wax dipping, embedding, sectioning at 5 μm thickness, and hematoxylin and eosin (H & E) staining. Liver morphology was examined under 10× and 40× objective lenses, and pathological changes were quantitatively evaluated based on the pathological scoring results. Images were randomly selected from each animal, and the histopathological scores were independently assessed by an investigator blinded to group allocation. Representative images and corresponding quantitative scores are presented in the relevant figures, with scale bars indicated in the figure legends.

### Immunofluorescence detection

The samples were completely covered with 5% goat serum for blocking, and the sections were placed in a humidified chamber and incubated at 37 °C for 30 min. The blocking solution was then removed, and the sections were incubated with the diluted primary antibodies anti-F4/80 (Cell Signaling Technology, Cat. No. 70076, 1:200), anti-Ly6G (Cell Signaling Technology, Cat. No. 87048, 1:200), and anti-CD8 (Cell Signaling Technology, Cat. No. 98941, 1:200) at 4 °C overnight. The sections were allowed to equilibrate to room temperature for 15 min and washed with TBST three times for 5 min each. The secondary antibody (Cell Signaling Technology, Cat. No. 4412, 1:500) was then applied and incubated at room temperature for 1 h in the dark. The sections were washed with TBST three times for 5 min each. DAPI working solution was added and incubated at room temperature for 10 min in the dark. After removal of the DAPI solution, the sections were washed with TBST three times for 5 min each, mounted with an anti-fade mounting medium, and examined under a fluorescence microscope. For each mouse, multiple randomly selected non-overlapping fields were imaged using the same acquisition settings. Images were randomly selected, and immunofluorescence-positive cells were quantified by an investigator blinded to group allocation using ImageJ software. Quantitative analysis was performed using individual mice as the biological replicates.

### Hepatocyte apoptosis detected by TdT-mediated dUTP nick-end labeling (TUNEL) staining

The frozen slices were fixed in 4% paraformaldehyde at room temperature for 25 min and washed three times with PBS for 5 min each. Samples were permeabilized with 1% Triton X-100 at room temperature for 5 min and washed three times with PBS. The TdT enzyme reaction mixture was prepared by adding 45 μL Equilibration buffer, 1 μL biotin-11-dUTP, and 4 μL TdT Enzyme. Samples were incubated with 50 μL of the reaction mixture at room temperature in a humidified chamber for 60 min, followed by three washes with PBS. Streptavidin-TRITC labeling solution was then applied for 30 min at room temperature in the dark, followed by three washes with PBS. The nuclei were stained with 1 mg/mL DAPI for 5 min, washed three times with PBS, and mounted with an anti-fade mounting medium. The TUNEL assay was performed using a commercial kit (KeyGEN BioTECH, Nanjing, China; Cat. No. KGA702) according to the manufacturer’s instructions, with appropriate positive and negative controls. For each mouse, multiple randomly selected non-overlapping fields were imaged using identical acquisition settings. TUNEL-positive cells were quantified using ImageJ and expressed as the percentage of TUNEL-positive nuclei among total DAPI-positive nuclei. Quantification was performed by an investigator blinded to group allocation, with individual mice used as biological replicates.

### TNF-α, CCL2 and CXCL5 were detected by ELISA

The mouse serum prepared from each group was subjected to TNF-α, CCL2, and CXCL5 ELISA (Elabscience). Following the instructions, 50–100 μL of sample was added to each reaction well. The mixture was incubated at 37 °C for 0.5∼1 h and then washed. With an enzyme-linked immunosorbent assay (ELISA) detector, the absorbance (OD) value of each well was detected after the blank control well was adjusted to zero at 450 nm.

### AST, ALT, SOD, CAT, GSH and GSH-Px measurements

The prepared liver tissue was mixed with precooled sodium chloride solution and fully prepared as a 10% tissue homogenate at low temperature with high-speed grinding. The prepared liver tissue homogenate was centrifuged at 4 °C at 4,000 rpm for 10 min, and the supernatant was removed for detection. Serum ALT and AST levels were measured using commercial assay kits (Nanjing Jiancheng Bioengineering Institute, Nanjing, China; ALT, C009-2-1; AST, C010-2-1) according to the manufacturer’s instructions. SOD, CAT, and GSH-Px activities in liver tissue homogenates were measured using commercial assay kits (Nanjing Jiancheng Bioengineering Institute, Nanjing, China; SOD, A001-3; CAT, A007-1; GSH-Px, A005) according to the manufacturer’s instructions.

### Cell culture

The Hepa1-6 mouse hepatoma cell line (CL-0105) was purchased from Wuhan Punose Life Technology Co., LTD. This cell line is derived from the C57L mouse strain and is widely employed in hepatocellular carcinoma research, particularly in immunocompetent C57BL/6 mouse models. Hepa1-6 cells were cultured in RPMI-1640 medium supplemented with 10% fetal bovine serum, 100 U/mL penicillin, and 100 μg/mL streptomycin at 37 °C with 5% CO_2_. After the cells in the logarithmic growth phase were digested with 0.25% trypsin, the cells were washed twice with serum-free RPMI-1640 medium and used for subsequent *in vitro* experiments. Hepa1-6 cells were treated with 0–160 μg/mL SMP for concentration screening. For overexpression experiments, cells were transfected with NIK or TRAF3 overexpression plasmids or the corresponding empty vector using Lipofectamine 3000 (Thermo Fisher Scientific, United States) according to the manufacturer’s instructions. After 24 h of transfection, the subgroups were oe-vector, oe-NIK plus SMP 10 μg/mL, oe-NIK plus SMP 20 μg/mL and oe-NIK plus SMP 40 μg/mL. For TRAF3 overexpression experiments, cells were divided into oe-vector and oe-TRAF3 groups. All cell-based experiments were performed using three independent biological replicates (n = 3).

### CCK-8

An enhanced cell viability test kit (CCK-8) (E-CK-A362, purchased from Elabscience) was used. The cell density was adjusted to 1 × 10^5^ cells/mL. Following treatment with the indicated concentrations of SMP, CCK-8 solution was added to each well and incubated at 37 °C according to the manufacturer’s instructions. The absorbance values of each group were measured at 450 nm using an automatic microplate reader, and cell viability was calculated according to the following formula:

$$\text{Cell viability }\left(\%\right)=\left(\text{experimental group }A\text{ value}/\text{control group }A\text{ value}\right)\times 100\%.$$

### Luciferase reporter assay

The NF-κB luciferase reporter plasmid was purchased from Promega. NIK overexpression vectors were cotransfected with luciferase reporter vectors into cells according to the manufacturer’s methods. Luciferase activity was detected using a dual luciferase reporter analysis system according to the manufacturer’s instructions (Promega).

### Western blot

The remaining liver tissue was collected, and an appropriate amount of RIPA lysis buffer was added to extract total protein. The protein concentration was determined using a BCA protein assay kit. For SDS-PAGE, proteins were separated at a constant voltage of 75 V through the stacking gel and 120 V through the resolving gel. The separated proteins were transferred to PVDF membranes at 100 V for 60 min. The membranes were blocked with 5% skimmed milk in TBST for 2 h at room temperature. Then, primary antibodies against NIK (#55247-1-AP, Proteintech), p52 (#15503-1-AP, Proteintech), NRF2 (#80593-1-RR, Proteintech), GAPDH (#60004-1-Ig, Proteintech), and TRAF3 (#SAB3500376-100UG, Sigma-Aldrich) were added at a dilution of 1:2000 and incubated ove,rnight at 4 °C. The membranes were washed three times with TBST for 10 min each and incubated with the appropriate secondary antibody (1:5,000) for 2 h at room temperature. After three washes with TBST for 10 min each, the membranes were incubated with ECL substrate for 1–2 min and imaged using a chemiluminescence imaging system. Band intensities were quantified using ImageJ software and normalized to GAPDH. For each experiment, protein expression was quantified from independent biological replicates, and the same exposure and analysis settings were used for all groups within an experiment.

For the NIK protein half-life assay, cells were treated with cycloheximide (CHX; 80 μM; #66-81-9, MedChemExpress, United States), and proteins were collected at 0, 2, 4, 6, and 8 h after treatment. Western blotting was then performed as described above.

### Co-IP and ubiquitinated IP assay

Coimmunoprecipitation (co-IP) experiments were conducted to elucidate the interaction between NIK and TRAF3. Briefly, cell lysates containing 100 μg of protein were subjected to immunoprecipitation with 5 μg of anti-NIK antibody. After an overnight incubation at 4 °C, 30 μL of protein A agarose bead mixture was added to each sample, which was then gently rocked for 3 h at 4 °C. The samples were subsequently centrifuged for 30 s and washed, and 20 μL of SDS buffer was added before heating at 100 °C for 5 min. A 30 μL aliquot of each sample was loaded onto a 10% SDS‒PAGE separating gel. After electrophoresis, the resolved proteins were transferred to PVDF membranes, blocked, and probed with an anti-TRAF3 primary antibody for 12 h at 4 °C. The signal was visualized using the electrochemiluminescence method. For the ubiquitination assay, Hepa1-6 cells were transfected with hemagglutinin-tagged ubiquitin (HA-Ub, #pCDNA3.1-HA-Ub-K48, Shanghai Jingfeng Biotechnology Co., Ltd., China). Following transfection, cells were treated with the proteasome inhibitor MG132 (#133407-82-6, MedChemExpress, United States). Cells were then lysed, and TRAF3 was immunoprecipitated from the cell lysates. The immunoprecipitated proteins were subjected to SDS‒PAGE and Western blotting, and ubiquitinated proteins were detected using an anti-HA antibody.

### qPCR (quantitative real-time PCR)

Total RNA was isolated from collected cells, reverse-transcribed into cDNA, and stored for subsequent qPCR analysis. The reaction mixture (20 μL) was prepared as follows: 5 μL of cDNA template, 8 μL of 2 × SYBR Green qPCR Master Mix, 0.4 μL of 50×Rhodamine-X (ROX), 0.4 μL of forward primer (10 μM), 0.4 μL of reverse primer (10 μM), and 5.8 μL of double-distilled water (ddH_2_O). After 30 s of pre-denaturation at 95 °C, 40 cycles of 5 s of denaturation at 95 °C and 30 s of annealing at 60 °C were performed. A melting-curve analysis was performed at the end of amplification. The relative quantitative results of the target genes of each sample were calculated and analyzed using the 2^−ΔΔCT^ formula.

**Table:** 

Primer	Sequence (5′-3′)
ACTIN F	GGC​TGT​ATT​CCC​CTC​CAT​CG
ACTIN R	CCA​GTT​GGT​AAC​AAT​GCC​ATG​T
TNF-α F	CCT​CTC​TCT​AAT​CAG​CCC​TCT​G
TNF-α R	GAG​GAC​CTG​GGA​GTA​GAT​GAG
CCL2 F	CAG​CCA​GAT​GCA​ATC​AAT​GCC
CCL2 R	TGG​AAT​CCT​GAA​CCC​ACT​TCT
CCL5 F	CCA​GCA​GTC​GTC​TTT​GTC​AC
CCL5 R	CTC​TGG​GTT​GGC​ACA​CAC​TT
CXCL5 F	AGC​TGC​GTT​GCG​TTT​GTT​TAC
CXCL5 R	TGG​CGA​ACA​CTT​GCA​GAT​TAC
NOS2 F	TTC​AGT​ATC​ACA​ACC​TCA​GCA​AG
NOS2 R	TGG​ACC​TGC​AAG​TTA​AAA​TCC​C

### DNA extraction, 16S rRNA gene sequencing, library construction and diversity analysis

A subset of mice (n = 5 per group, total 15 mice) from the *in vivo* animal experiment were selected for 16S rRNA gene sequencing and untargeted metabolomics analysis of cecal contents. DNA was isolated from the cecal contents using a DNA extraction kit (#DP328, TIANGEN BIOTECH CO., Ltd., Beijing, China). For library construction, the primers 341F (CCTACGGGNGGCWGCAG) and 805R (GACTACHVGGGTATCTAATCC) were used to amplify the V3 and V4 regions of the 16S rRNA gene. The genomic DNA of the mouse intestinal microbiome was amplified, standardized and sequenced using Illumina MiSeq sequencers (2 × 300 bp double-terminal run). After nonbiological sequences were removed, the raw data were quality-filtered using QIIME2 software (https://qiime2.org/) and processed using the DADA2 pipeline to generate amplicon sequence variants (ASVs). Specific DADA2 parameter settings were strictly defined as follows: the maximum expected error (maxEE) was set to 2 for both forward and reverse reads; forward reads were truncated at 280 bp and reverse reads at 220 bp. Subsequent dereplication, denoising, and chimera removal were performed following standard DADA2 workflows. The resulting ASVs were used for downstream diversity and taxonomic analyses. To eliminate the bias of uneven sequencing depth across samples, all samples were rarefied to a uniform depth of 20,000 sequences per sample prior to α-diversity, β-diversity (PCoA), and differential abundance analysis. Taxonomic classification was performed using the RDP naïve Bayes classifier with the SILVA reference database (Release 132). The calculation of alpha diversity was performed with the Vegan package in R software. β diversity analysis was based on the weighted UniFrac distance and was performed via principal coordinate analysis (PCoA). Group differences in β-diversity were assessed using ANOSIM LDA effect size analysis (LEfSe) was conducted to screen the marker taxa from each group. Functional prediction and pathway analysis were performed using PICRUSt2 and the Kyoto Encyclopedia of Genes and Genomes (KEGG) database.

### Untargeted metabolomics

For the untargeted metabolomics study conducted using liquid chromatography‒mass spectrometry (LC‒MS) on cecal contents stored at −80 °C, the samples were initially thawed and homogenized. Approximately 50 mg of each sample was mixed with 500 μL of pre-cooled methanol/acetonitrile (1:1, v/v) and vortexed for 30 s. Samples were incubated at −20 °C for 60 min, then centrifuged at 12,000 × *g* for 15 min at 4 °C. The clear supernatants were collected for subsequent LC-MS analysis. Chromatographic separation was performed using an ultrahigh-performance liquid chromatography (UHPLC) system coupled to a high-resolution mass spectrometer operated in both positive- and negative-ion modes. Full-scan data were acquired over an m/z range of 100–1,500. Quality control (QC) samples were included to monitor analytical stability, and blank samples were used to identify background signals. The resulting chromatographic data were processed for peak detection, alignment, and metabolite annotation. After peak detection and alignment, total ion current (TIC) normalization was applied to standardize metabolomic data and reduce technical variation before principal component analysis (PCA) and differential metabolite abundance testing. Missing values were handled before statistical analysis, and multiple comparisons were controlled using the Benjamini–Hochberg false discovery rate (FDR) procedure. Differential metabolite features were defined based on fold change >1.2, VIP > 1, and FDR-adjusted P < 0.05. The reported metabolites were considered annotated features rather than fully confirmed metabolites unless supported by authentic standards and retention-time matching. Pathway enrichment analysis of the differentially abundant metabolites was performed via the KEGG database.

### Statistical analysis

Statistical analyses were performed using SPSS 25.0 and R software. Quantitative data are presented as the mean ± SD. Data normality and homogeneity of variance were assessed before parametric analyses. For comparisons between two groups, a two-tailed Student’s t-test was used. For comparisons among multiple groups, one-way analysis of variance (ANOVA) followed by the Student–Newman–Keuls *post hoc* test was used. For body weight and fasting blood glucose measurements in the toxicity assessment, differences among groups were analyzed independently at each measurement time point. Categorical data were analyzed using the chi-square test. Correlations between gut microbiota and metabolites were assessed using Spearman’s rank correlation analysis. For microbiome and metabolomics analyses, P values from multiple comparisons were adjusted using the Benjamini–Hochberg false discovery rate (FDR) procedure, and FDR-adjusted P values were reported. Exact P values were reported where applicable. For non-omics analyses, *P* < 0.05 was considered statistically significant, whereas an FDR-adjusted *P* < 0.05 was considered statistically significant for microbiome and metabolomics analyses.

## Results

### Toxicity testing of SMP in mice

Adult male C57BL/6J mice (8 weeks old) were administered 200 mg/kg SMP daily via oral gavage for 14 days (SMP group), while the control group received an equivalent volume of normal saline. No significant differences were observed between the two groups in terms of body weight, fasting blood glucose levels, or liver and spleen wet weights (*P* > 0.05, Figures 1A–C). Furthermore, SMP prevention did not alter serum ALT activity (*P* > 0.05, Figure 1D), indicating no hepatotoxic effects. Collectively, these findings demonstrate that SMP administration at the tested dose was well tolerated and did not induce any detectable toxicological side effects in mice.

**FIGURE 1:**
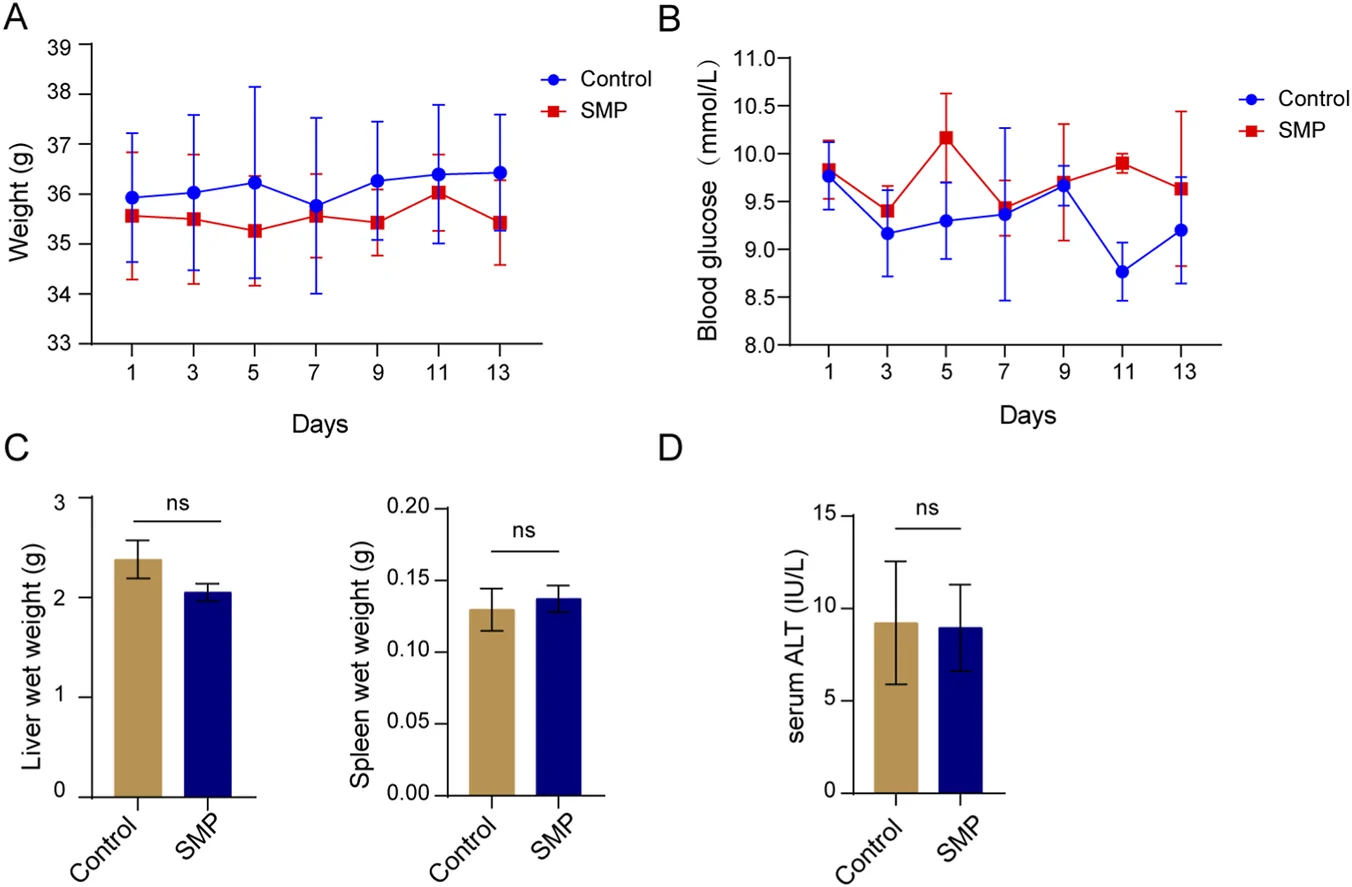
Toxicity test of SMP in mice. After 2 weeks of drug intervention (13th day), fresh blood samples from the mice were obtained for aminotransferase activity analyses. **(A)** The body weights of the mice were recorded during the trial; **(B)** The daily fasting blood glucose values of the mice were recorded during the trial. **(C)** Wet weights of the livers and spleens of the mice at the end of the trial; **(D)** Colorimetry-based serum aminotransferase activity at the end of the trial. All the data are presented as the means ± SDs. n = 8. For body weight and fasting blood glucose, differences among groups were analyzed independently at each measurement time point using one-way ANOVA followed by the Student–Newman–Keuls *post hoc* test. For liver and spleen weights and serum aminotransferase activities, one-way ANOVA followed by the Student–Newman–Keuls *post hoc* test was used. SMP, Silybum marianum peptides.

### SMP protected against CCl_4_-induced liver injury in mice

As shown in Figure 2A, neither CCl_4_ nor SMP significantly affected the liver or spleen indices of the mice. Additionally, CCl_4_ induced cytoplasmic vacuolation, increased nuclear condensation, disordered arrangement, and massive infiltration of inflammatory cells in the mouse liver, which demonstrated successful liver injury in the mice (*P* < 0.05, Figure 2B). SMP at doses of 50, 100 and 200 mg/kg significantly alleviated CCl_4_-induced liver injury in mice (*P* < 0.05, Figure 2B). CCl_4_ induced an imbalance in red and white pulp distribution and white pulp atrophy, hemorrhage, and necrosis in the mouse spleen, but SMP did not significantly prevent CCl_4_-induced spleen injury in mice (Figure 2B). Among the three tested doses, SMP at 200 mg/kg significantly reversed the elevated hepatocyte apoptosis in CCl_4_-induced mice (*P* < 0.05, Figure 2C). At doses of 100 and 200 mg/kg, SMP markedly suppressed CCl_4_-induced elevations in proinflammatory cytokines TNF, CXCL5 (*P* < 0.05, Figure 2D) and serum transaminases AST, ALT (*P* < 0.05, Figure 2E), while it exerted no significant effect on CCL2 levels (Figure 2E). Additionally, 200 mg/kg SMP restored hepatic antioxidant capacity by reversing CCl_4_-induced declines in SOD and GSH-Px activities (*P* < 0.05, Figure 2F).

**FIGURE 2:**
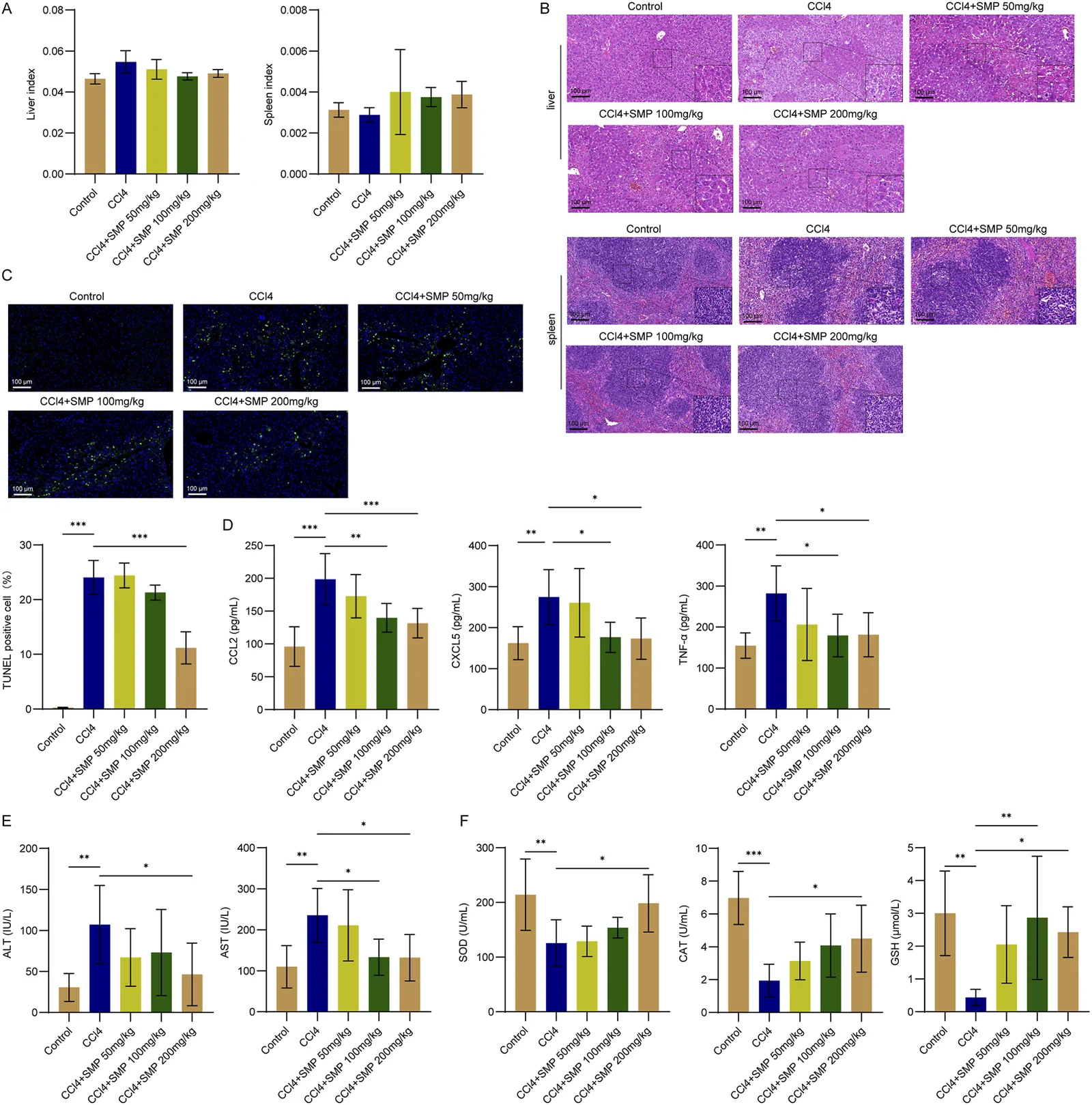
Protective effects of SMP against CCl_4_-induced acute liver injury. 8-Week-old male C57BL/6J mice were divided into the following five groups: the control group (treated daily with saline and an intraperitoneal injection of olive oil on the 14th day), CCl_4_ group (treated daily with saline and an intraperitoneal injection of CCl_4_ dissolved in olive oil on the 14th day), SMP group (treated daily with SMP at 50, 100, or 200 mg/kg, and an intraperitoneal injection of CCl_4_ dissolved in olive oil on the 14th day). **(A)** Organ indices of the liver and spleen; **(B)** Pathological damage to the liver and spleen was assessed via H & E staining and microscopic observation. **(C)** The number of apoptotic cells in liver tissue was determined via TUNEL staining; **(D)** Cytokine levels, including those of TNF-α, CCL2, and CXCL5, were detected by ELISA assays; **(E)** Aminotransferase AST and ALT activities were detected by colorimetric assays; **(F)** Antioxidant indicators of the liver include SOD activity, CAT activity, and GSH content. All data are presented as the mean ± SD. Statistical significance was determined by one-way ANOVA followed by the Student–Newman–Keuls *post hoc* test. n = 8, *P < 0.05, **P < 0.01, ***P < 0.001.

Taken together, these findings demonstrate that SMP effectively mitigates CCl_4_-induced hepatic damage in mice, with 200 mg/kg being the most effective of the tested doses for hepatoprotection.

### SMP protected against CCl_4_-induced liver injury in mice by inhibiting NIK/NF-κB

Compared with those in the control group, the numbers of macrophages (F4/80), neutrophils (Ly6G), and CD8 T cells (CD8^+^) in the liver tissues of the CCl_4_ group were significantly greater (*P* < 0.001, Figures 3A,B), whereas the 200 mg/kg SMP significantly reduced CCl_4_-induced macrophage, neutrophil and CD8 T-cell (CD8^+^) infiltration (*P* < 0.01, Figures 3A–C). In addition, the 200 mg/kg SMP reversed the CCl4-induced decreasing expression of Nrf2 and TRAF3 (*P* < 0.05, Figures 3D,E). And decreased the expression of NIK and p52 in CCl_4_-induced mouse livers (*P* < 0.05, Figure 3F; Supplementary Figure S2). These findings suggest that SMP may protect against CCl_4_-induced liver injury, at least in part, through modulation of immune cell infiltration and the NIK/NF-κB pathway.

**FIGURE 3:**
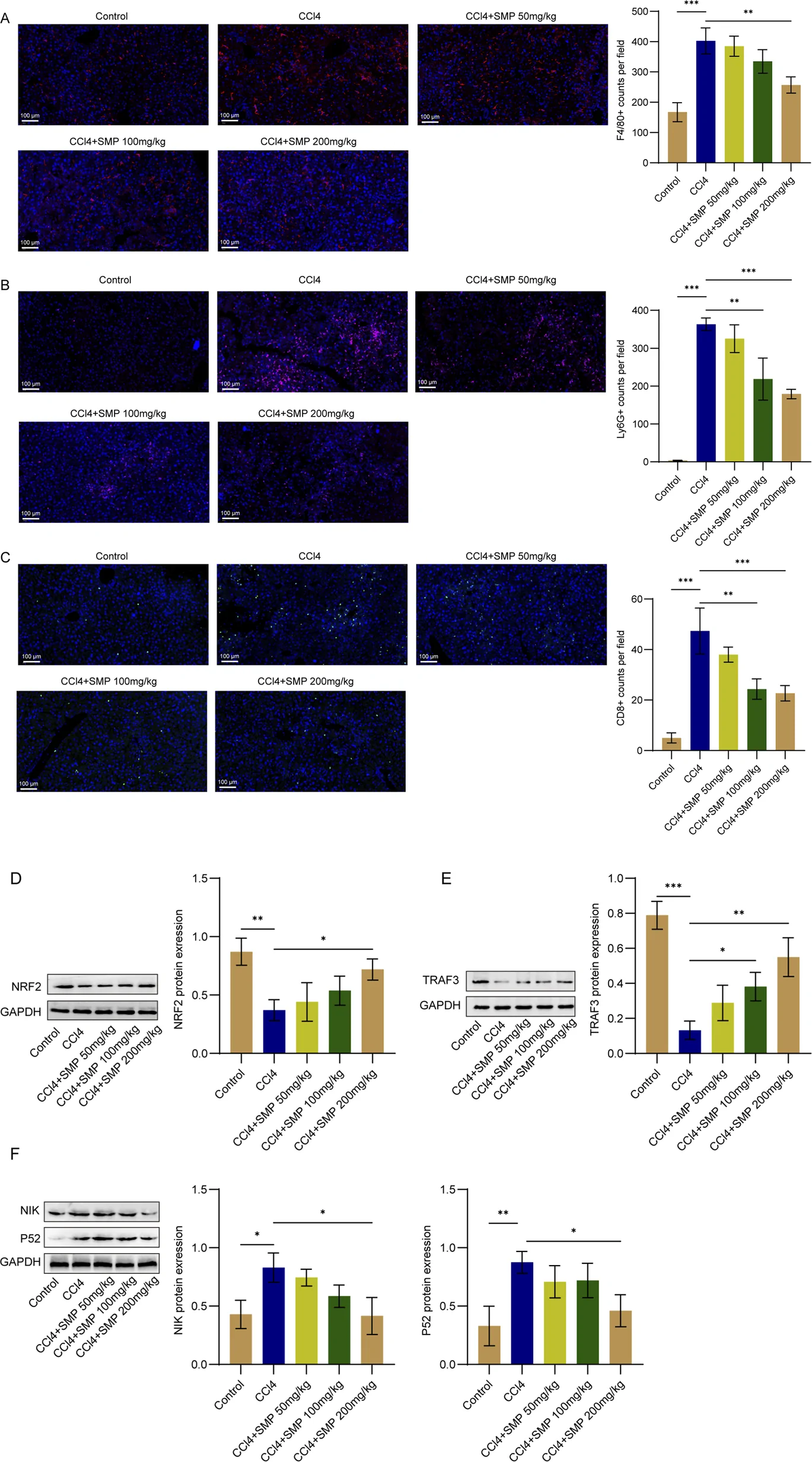
SMP protected against CCl_4_-induced liver injury in mice by inhibiting NIK/NF-κB. 8-Week-old male C57BL/6J mice were divided into the following five groups: the control group (treated daily with saline and an intraperitoneal injection of olive oil on the 14th day), CCl_4_ group (treated daily with saline and an intraperitoneal injection of CCl_4_ dissolved in olive oil on the 14th day), SMP group (treated daily with SMP at 50, 100, or 200 mg/kg, and an intraperitoneal injection of CCl_4_ dissolved in olive oil on the 14th day). **(A)** Immunofluorescence observation of macrophages in liver tissue (F4/80); **(B)** Immunofluorescence observation of neutrophils in liver tissue (Ly6G); **(C)** Immunofluorescence observation of CD8 T cells in liver tissue (CD8^+^); **(D)** Protein expression levels of NRF2 determined via Western blot; **(E)** Protein expression levels of TRAF3 determined via Western blot; **(F)** Protein expression levels of NIK, and p52 determined via Western blot. All data are presented as the mean ± SD. Statistical significance was determined by one-way ANOVA followed by the Student–Newman–Keuls *post hoc* test. For immunofluorescence and Western blot analyses, quantitative data were derived from independent biological samples from individual mice. n = 8, *P < 0.05, **P < 0.01, ***P < 0.001.

### SMP promoted NIK ubiquitination and was associated with TRAF3 expression

The expression of NIK protein was significantly downregulated in TRAF3-overexpressing (oe-TRAF3) cells, mirroring the effect observed in SMP-treated cells (P < 0.05, Figure 4A; Supplementary Figure S3A). Co-immunoprecipitation (co-IP) analysis supported an association or co-complex formation between NIK and TRAF3 (Figure 4B; Supplementary Figure S3B). Cycloheximide (CHX) chase experiments revealed that both SMP treatment and TRAF3 overexpression significantly accelerated NIK protein degradation, with the most pronounced effect observed at 8 h (*P* < 0.05, Figure 4C; Supplementary Figure S3C). Furthermore, ubiquitination assays demonstrated that SMP prevention or TRAF3 overexpression markedly enhanced NIK protein ubiquitination (*P* < 0.05, Figure 4D; Supplementary Figure S3D). These findings suggest that SMP may promote NIK ubiquitination and degradation in association with TRAF3, although the specific ubiquitin linkage and the causal dependence of the SMP effect on TRAF3 remain to be determined.

**FIGURE 4:**
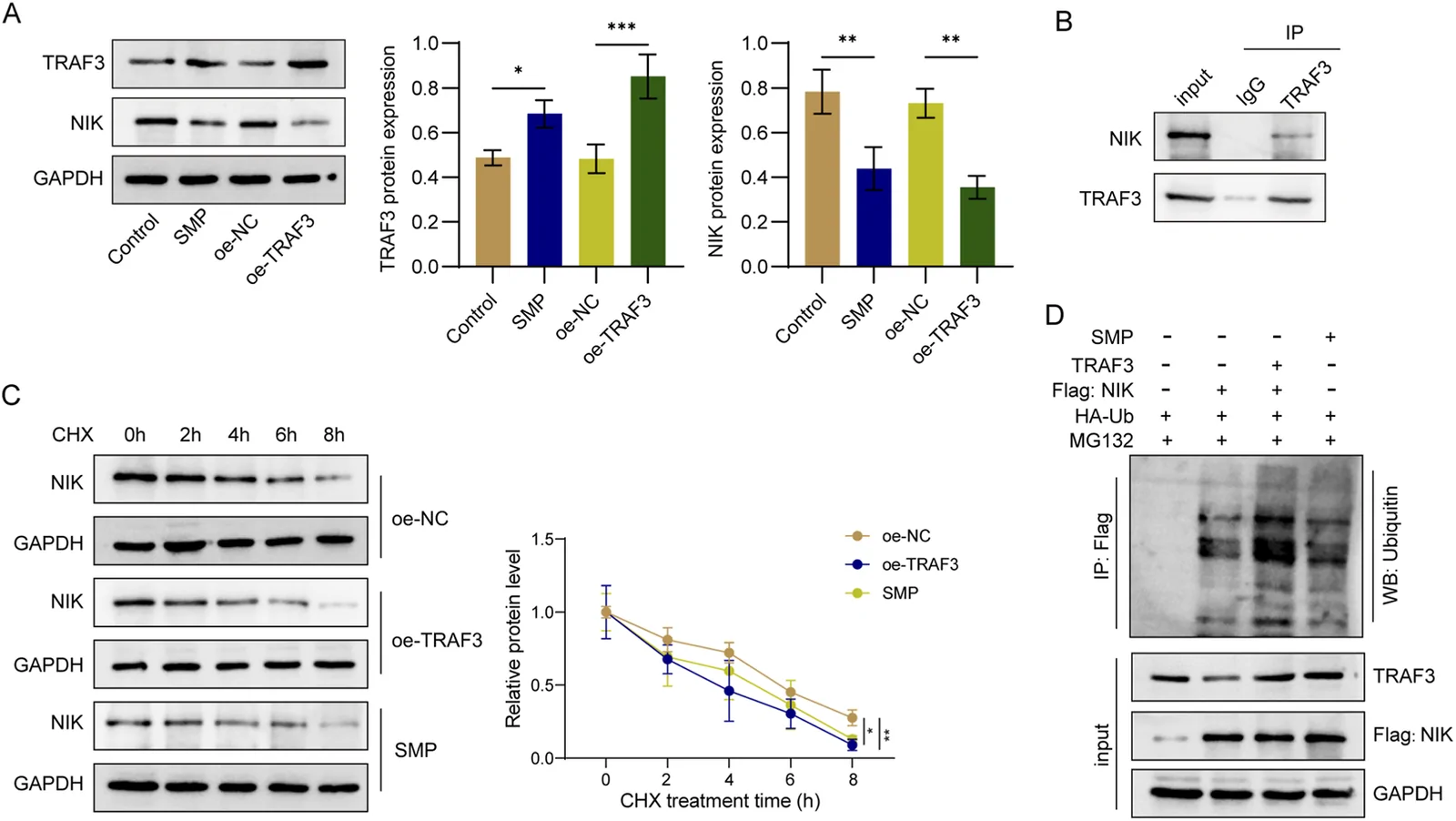
SMP-associated NIK ubiquitination in association with TRAF3. Hepa1-6 cells were divided into 4 treatment groups: the control group (normal cells), SMP group (cells treated with 40 μg/mL), oe-NC group (cells overexpressing the vector), and oe-TRAF3 group (cells overexpressing TRAF3). **(A)** Protein expression levels of NIK and TRAF3 determined via Western blot; **(B)** The binding of the NIK and TRAF3 proteins was detected via a coimmunoprecipitation assay. **(C)** The half-life of the NIK protein was detected after different durations of CHX treatment. **(D)** The level of NIK ubiquitination was analyzed after proteasome inhibitor (MG132) treatment. Data are presented as the mean ± SD from three independent experiments (n = 3), with three technical replicates per independent experiment. Statistical significance was determined by one-way ANOVA followed by the Student–Newman–Keuls *post hoc* test. *P < 0.05, **P < 0.01, and ***P < 0.001.

### SMP alleviated cell damage by inhibiting NIK/NF-κB

SMP significantly reduced the Hepa1-6 cells viability at a concentration of 160 μg/mL, which was similar to the cytotoxic concentration of silymarin reported in a previous study (Sainz-Pardo et al., 1994); thus, lower concentrations of 10, 20, and 40 μg/mL were selected for subsequent experiments (Figure 5A). SMP at 40 μg/mL significantly reversed the increased protein expression of NIK and p52 in oe-NIK Hepa1-6 cells (*P* < 0.01, Figure 5B) and significantly reversed the decreased Nrf2 protein expression in oe-NIK Hepa1-6 cells (*P* < 0.01, Figure 5B; Supplementary Figure S4). The relative luciferase activity of the Hepa1-6 cells in the oe-NIK group was greater than that in the oe-NC group (*P* < 0.001, Figure 5C), indicating enhanced transcriptional activity, whereas 40 μg/mL SMP significantly reduced the relative luciferase activity of oe-NIK cells (*P* < 0.01, Figure 5C). Moreover, compared with oe-NC Hepa1-6 cells, oe-NIK Hepa1-6 cells presented greater expression of TNF-α, CCl2 and CCl5 (*P* < 0.01, Figures 5D,E), whereas 40 μg/mL SMP inhibited the mRNA expression of TNF-α, CCL2 and CCL5 and downregulated the concentrations of TNF-α and CCL2 (*P* < 0.05, Figures 5D,E). Furthermore, 40 μg/mL SMP reversed the increase in the expression of the CXCL5 and NOS2 mRNAs in oe-NIK Hepa1-6 cells (Figure 5F). Collectively, these findings demonstrate that SMP effectively inhibits the NIK/NF-κB signaling pathway in NIK-overexpressing Hepa1-6 cells, highlighting its *in vitro* potential as a preventive hepatoprotective agent related to acute liver injury.

**FIGURE 5:**
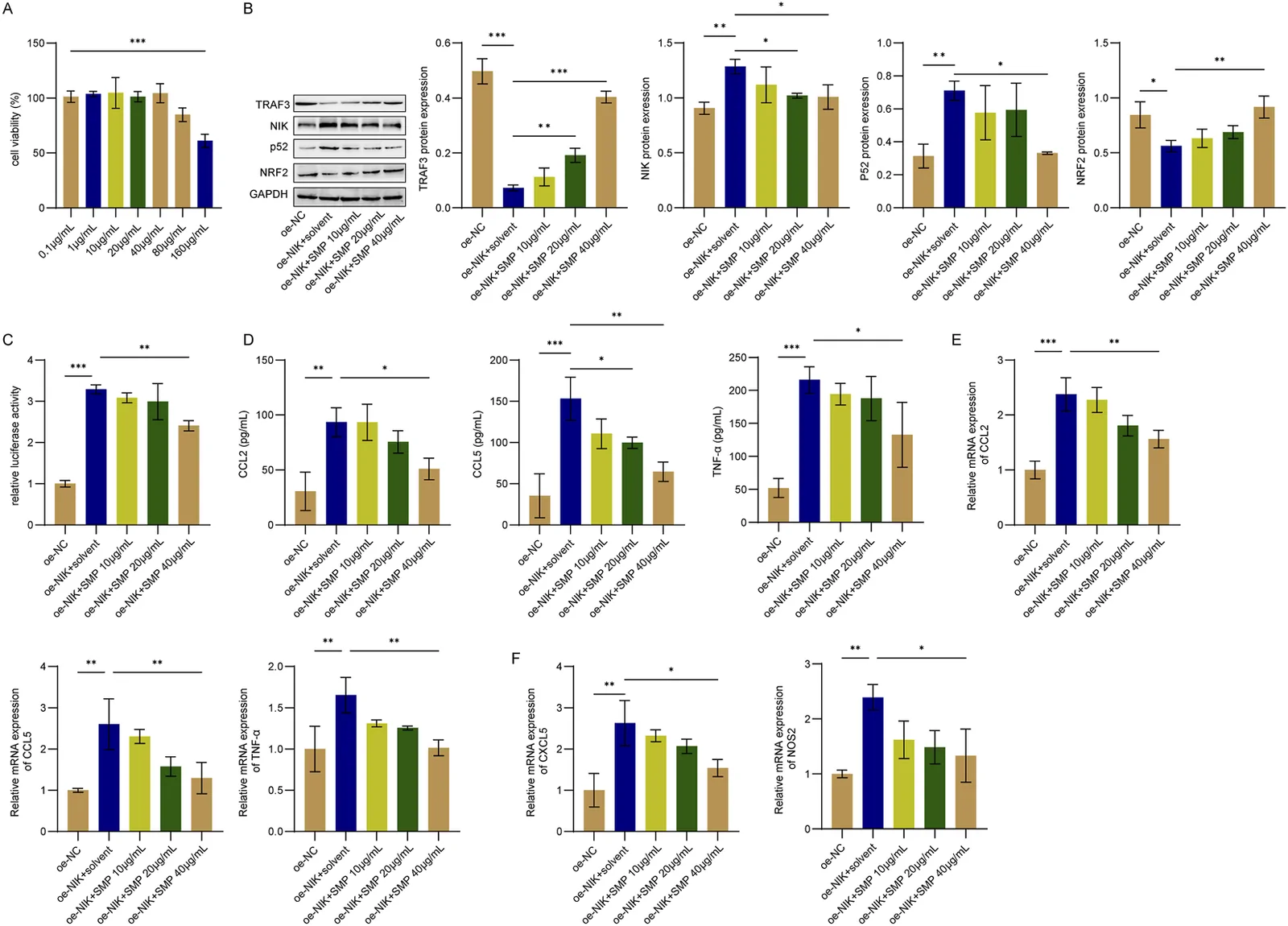
SMP alleviated cell damage by inhibiting NIK/NF-κB. After the appropriate SMP concentration (0, 0.01, 0.1, 1, 10, 20, 40, 80, or 160 μg/mL) was determined in normal Hepa1-6 cells, the effect of SMP on the expression of inflammation-induced marker genes was evaluated in Hepa1-6 cells overexpressing the NIK gene. Hepa1-6 cells were divided into the following five groups (n = 3): the oe-vector group (overexpressing vector and administered saline), the solvent group (overexpressing the NIK gene and administered solvent), and the SMP group (overexpressing the NIK gene and administered SMP at concentrations of 10, 20, or 40 μg/mL). **(A)** CCK8 assay was used to measure the viability of normal Hepa1-6 cells; **(B)** Protein expression levels of NRF2, NIK, and p52 determined via Western blot; **(C)** Relative dual luciferase activity indicating transcription activity; **(D)** mRNA expression levels of TNF-α, CCL2, and CCL5, as detected by qPCR; **(E)** Cytokine levels, including those of TNF-α, CCL2, and CCL5, were detected via ELISA; **(F)** The mRNA expression levels of CXCL5 and NOS2 were detected by qPCR. Data are presented as the mean ± SD from three independent experiments (n = 3), with three technical replicates per independent experiment. Statistical significance was determined by one-way ANOVA followed by the Student–Newman–Keuls *post hoc* test. N = 3, *P < 0.05, **P < 0.01, ***P < 0.001. SMP, Silybum marianum peptides.

### Effects of SMP on cecal microbial diversity and taxonomic composition in CCl_4_-induced ALI mice

The α-diversity indices (Chao1, Shannon and Simpson) are displayed in Figure 6A. Both the CCl_4_ and SMP groups showed lower Chao1, Shannon, and Simpson indices and higher Good’s coverage than the solvent group, but without significant differences (Figure 6A). In β-diversity ordination, the SMP group was located nearer to the control group relative to the CCl_4_ group (Figures 6B,C). At the phylum level, Firmicutes and Bacteroidetes were predominant, with total relative abundances greater than 80% (Figure 6D). At the genus level, *Akkermansia*, *Alistipes*, *Lachnospiraceae_NK4A136_group*, *Helicobacter* and *Odoribacter* were the top 5 genera with a total relative abundance of approximately 40%. The most dominant genus in the CCl_4_ group and SMP group was *Alistipes*, while the most dominant genus in the control group (solvent) was *Akkermansia*. LEfSe analysis showed that relative abundances of *Alistipes*, *Colidextribacter*, *Prevotellaceae* UCG-001, *Oscillibacter* and unidentified *Rhodospirillales* were markedly elevated, while that of the genus *Bifidobacterium* declined significantly in the CCl_4_ group relative to the solvent control group (*P* < 0.05, t-test; Figure 6E). Among these altered taxa, *Alistipes* and *Prevotellaceae* UCG-001 have been associated with inflammatory conditions in previous studies, and the drop in *Bifidobacterium* further confirmed the intestinal toxicity induced by CCl_4_ prevention. The SMP group possessed remarkably lower relative abundances of *Akkermansia*, *Lachnoclostridium*, *Clostridium_sensu_stricto_1* and *Oscillibacter* when compared with the CCl_4_ group (*P* < 0.05, t-test; Figure 6E). Collectively, SMP was associated with shifts in the overall structure and composition of gut microbiota following CCl_4_ exposure.

**FIGURE 6:**
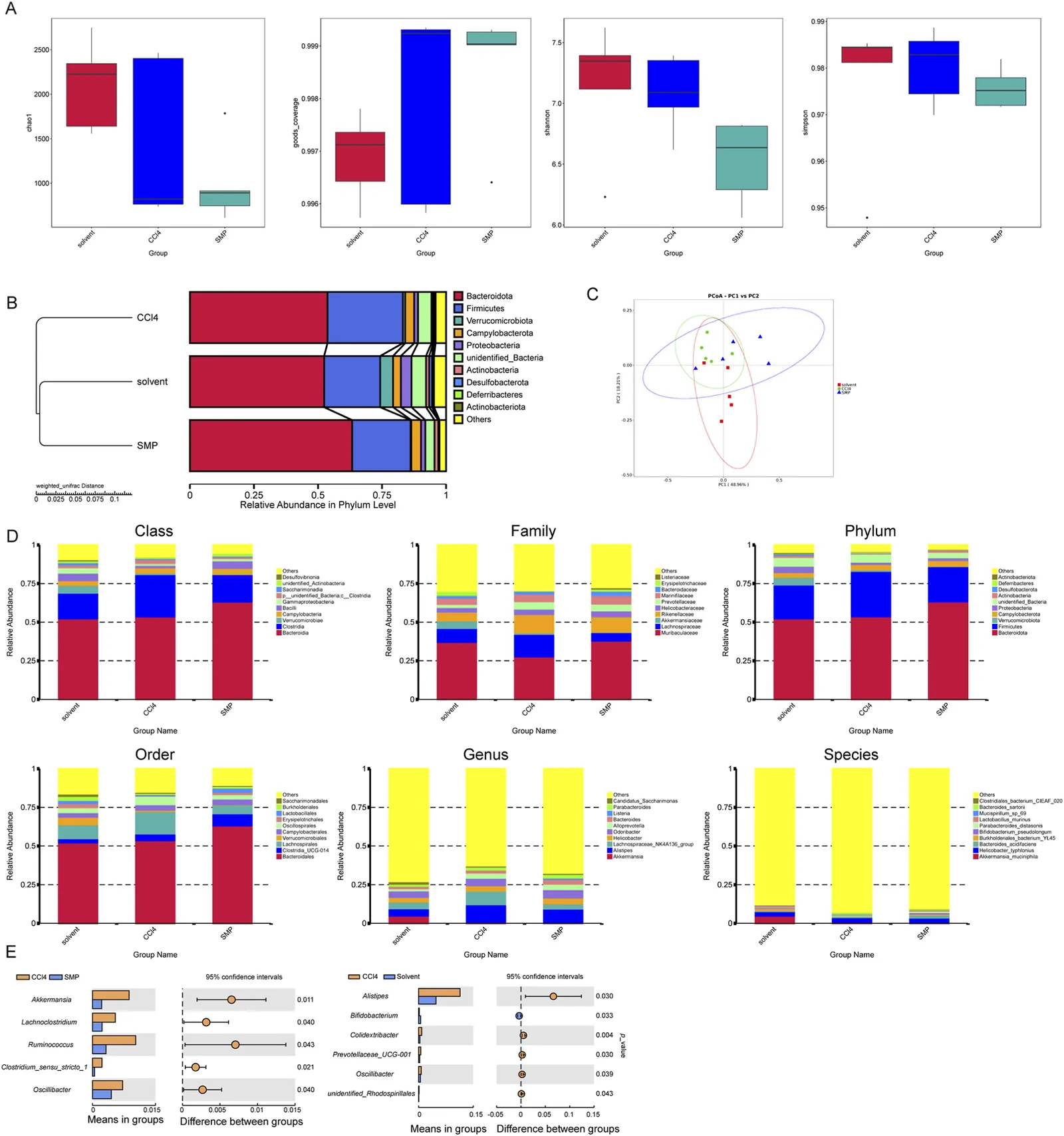
Effects of SMP on cecal microbial diversity and taxonomic composition in CCl_4_-induced ALI mice. A total of 15 8-week-old male C57BL/6J mice were divided into the following three groups (n = 5): the control group (treated daily with saline and an intraperitoneal injection of olive oil on the 14th day), the CCl_4_ group (treated daily with saline and an intraperitoneal injection of CCl_4_ dissolved in olive oil on the 14th day), and the SMP group (treated daily with 200 mg/kg, and an intraperitoneal injection of CCl_4_ dissolved in olive oil on the 14th day). **(A)** α diversity includes the Chao1, Shannon and Simpson indices; **(B)** β diversity based on weighted UniFrac distance, represented by cluster tree **(C)** PCoA, and discriminated between groups according to ANOSIM analysis; **(D)** Mean relative abundances at the phylum and genus taxonomic levels; **(E)** LEfSe analysis identifying differentially abundant taxa between the control and CCl_4_ groups and between the CCl_4_ and SMP-treated groups. For α-diversity comparisons, one-way ANOVA followed by the Student–Newman–Keuls *post hoc* test was used. β-diversity was evaluated using ANOSIM. Differential-abundance analyses were performed using LEfSe, with multiple testing controlled using the false discovery rate (FDR) procedure.

### Nontarget metabolomic analysis of mouse cecal contents

As shown in Figure 7A, a total of 278 differentially abundant metabolites were identified between the CCl_4_ group and the solvent control group, among which 237 were upregulated and 41 were downregulated. Between the SMP group and CCl_4_ group, 25 differential metabolites were detected, including 22 upregulated and 3 downregulated metabolites (fold change > 1.2, VIP > 1, *P* < 0.05). The fold changes in the differentially abundant metabolites in the top 20 are shown in Figure 7B. Compared with the control group, the CCl_4_ group presented higher levels of alpha-benzyl succinic acid, xanthurenic acid, 4-hydroxyretinoic acid, 1,2,3-cyclopropanetricarboxylic acid and meclocycline, and lower levels of pilocarpine, 2-oxindole, L(+)-citrulline, NZ-benzyldiene-S-hex-1-ynylfuran-2-carbohydrazide and 2-[(3,4,3,4,5,6-tetramethylphenyl) prop-2-ynyl]oxy] tetrahydro-2H-pyran. SMP significantly increased the levels of RMH, LPC 18:1,2-oxindole, LPI 18:2 and stachyose and significantly decreased the levels of alpha-farnesene, hydroxyprogesterone caproate and KM1H compared with those in the CCl_4_ group. KEGG pathway analysis (Figure 7C) demonstrated that DAMs between the CCl_4_ and control groups were enriched in steroid hormone biosynthesis, prostate cancer, and cancer-related pathways. In contrast, SMP-associated DAMs were primarily involved in galactose metabolism (P < 0.05).

**FIGURE 7:**
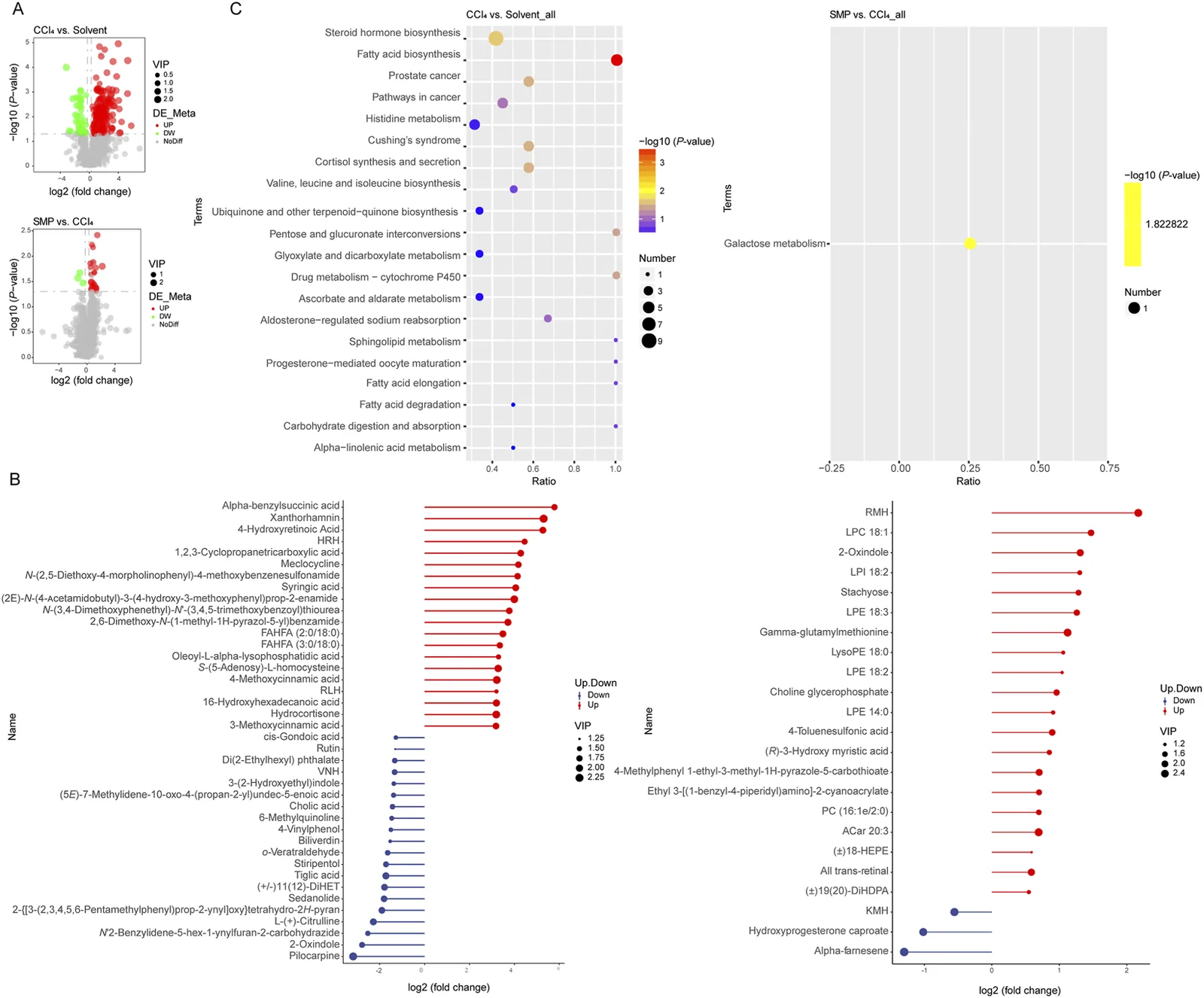
Effects of SMP on the cecal metabolite profile in CCl_4_-induced ALI mice. A total of 15 8-week-old male C57BL/6J mice were divided into the following three groups (n = 5): the control group (treated daily with saline and an intraperitoneal injection of olive oil on the 14th day), CCl_4_ group (treated daily with saline and an intraperitoneal injection of CCl_4_ dissolved in olive oil on the 14th day), and the SMP group (treated daily with 200 mg/kg, and an intraperitoneal injection of CCl_4_ dissolved in olive oil on the 14th day). **(A)** Volcano plots showing differential metabolites between the CCl_4_ and control groups and between the CCl_4_ and SMP-treated groups, setting the threshold to fold change (FC) > 1.2 or FC < 0.833 with p < 0.05, where the x-axis represents the log2FC and the y-axis represents the *P* value; **(B)** The top 20 differentially abundant metabolites between the control and CCl_4_ groups and between the CCl_4_ and SMP-treated groups; **(C)** KEGG pathway enrichment of differentially abundant metabolites (p < 0.05). Metabolomics analyses were considered exploratory, and multiple testing was controlled using the false discovery rate (FDR) procedure. SMP Silybum marianum peptides.

### Metabolite‒microbiota correlation analysis

Correlation analysis revealed exploratory associations between gut microbial taxa and host metabolites. For *Colidextribacter*, which was enriched in the CCl_4_ group, several metabolites exhibited significant correlations after FDR adjustment, including (+)-biotin N-hydroxysuccinimide ester, 3-methylindole, (2E)-N-(4-acetamidobutyl)-3-(4-hydroxy-3-methoxyphenyl) prop-2-enamide, and other phenolic and lipid-derived metabolites. In contrast, DL-citrulline showed a significant negative correlation with Colidextribacter and most of the aforementioned metabolites (Figure 8A).

**FIGURE 8:**
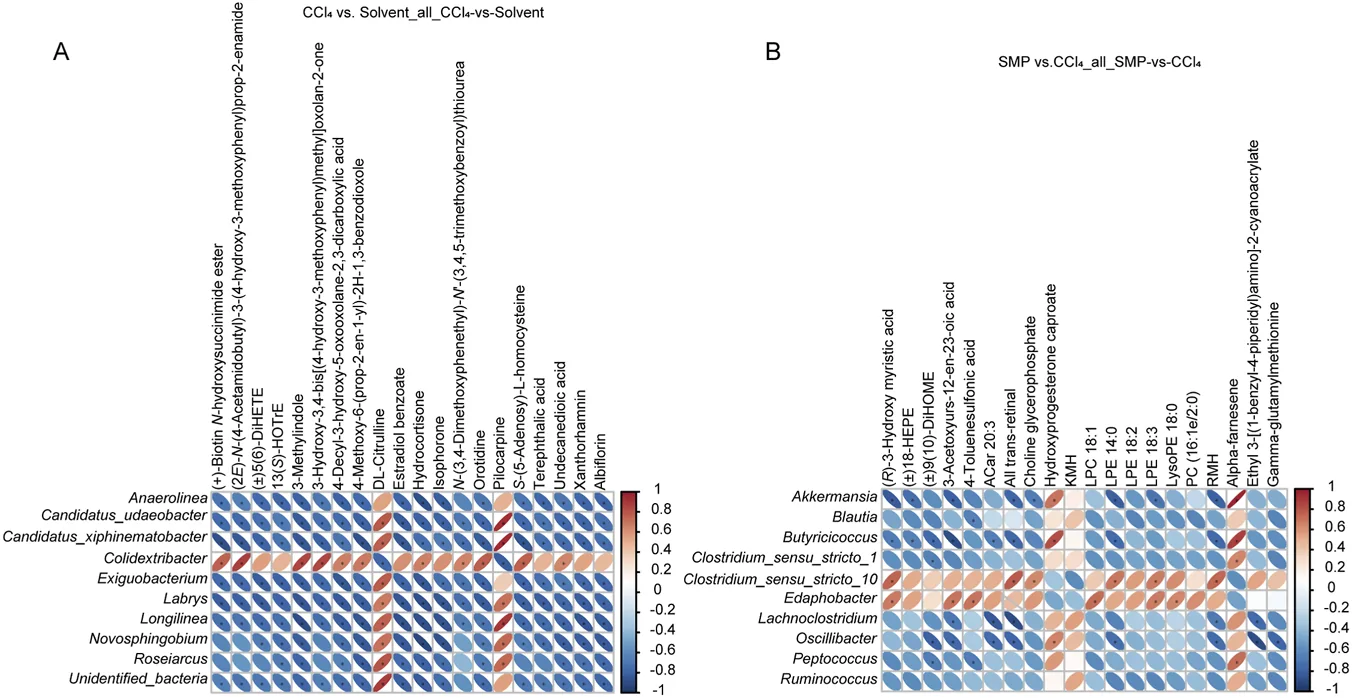
Association analysis of the gut microbiota and untargeted metabolomics in CCl_4_-induced ALI mice. A total of 15 8-week-old male C57BL/6J mice were divided into the following three groups (n = 5): the control group (treated daily with saline and an intraperitoneal injection of olive oil on the 14th day), CCl_4_ group (treated daily with saline and an intraperitoneal injection of CCl_4_ dissolved in olive oil on the 14th day), and SMP group (treated daily with 200 mg/kg, and an intraperitoneal injection of CCl_4_ dissolved in olive oil on the 14th day). **(A)** Spearman correlation of gut microbes and differentially abundant metabolites between the control group and the CCl_4_ group; **(B)** Spearman correlation of gut microbes and differentially abundant metabolites between the SMP group and CCl_4_ group. Spearman correlation coefficients were calculated using individual mouse-level data, and multiple comparisons were controlled using the false discovery rate (FDR) procedure. SMP, *Silybum marianum* peptides.

For *Akkermansia*, an inverse correlation was observed with multiple lipid- and inflammation-related metabolites, including (R)-3-hydroxymyristic acid, RMH, lysophospholipids (LPE 18:3, LPE 14:0, and LPI 18:2) (±)18-HEPE, all-trans-retinal, choline glycerophosphate, stachyose, and several other metabolites. Conversely, hydroxyprogesterone caproate and α-farnesene were positively associated with Akkermansia abundance (Figure 8B). These exploratory associations do not establish a causal role for Akkermansia in lipid metabolism or inflammatory responses during liver injury.

## Discussion

ALI represents a critical health challenge, often leading to severe liver dysfunction and potential mortality. The urgent need to develop effective prevention strategies for ALI is underscored by the complexity and severity of this condition (Björnsson and Björnsson, 2022; Norman, 2020). *Silybum marianum* is well known for its hepatoprotective effects, which are attributed primarily to the flavonoid complex silymarin (Yassin et al., 2022; Gültekin, 2023; Feng et al., 2016). Silymarin has shown beneficial effects in both preclinical models and clinical trials through its antioxidative, anti-inflammatory, and liver-protective properties. However, the detailed molecular mechanisms underlying its effects on liver injury remain unclear. Our study introduces a novel bioactive compound, SMP, which is more easily absorbed derived from *S. marianum* and investigates its potential as a protection agent for CCl_4_-induced ALI in mice. We found that SMP exerts significant hepatoprotective effects, reducing immune cell infiltration and the levels of proinflammatory cytokines and oxidative stress markers. These results suggest that SMP may represent a promising hepatoprotective candidate for preventing CCl_4_-induced liver injury.

Silymarin, the most extensively studied bioactive compound from *S. marianum*, exerts potent hepatoprotective effects in various liver diseases by reducing oxidative stress through ROS scavenging and modulating proinflammatory cytokines to alleviate inflammation (Jaffar et al., 2024; Aghemo et al., 2022; Gillessen and Schmidt, 2020). In a CCl_4_-induced ALI model, silymarin improved liver function by reducing the serum levels of ALT and AST, which are indicative of liver injury (Abdel-Moneim et al., 2015). Beyond silymarin, other bioactive compounds from *S. marianum*, especially silibinin, have demonstrated hepatoprotective properties by inhibiting hepatocarcinogenesis through the HGF/c-Met, Wnt/β-catenin, and PI3K/Akt/mTOR pathways (Yassin et al., 2022; Gültekin, 2023; Feng et al., 2016), and by mitigating ethanol- and acetaldehyde-induced liver injury via suppression of oxidative stress and mitochondrial fission (Song et al., 2022). In addition, advanced silymarin formulations, such as phytosomes and nanoparticles, showed enhanced efficacy in treating nonalcoholic fatty liver disease and fibrosis (Abdullah et al., 2022; Shriram et al., 2022). Our findings are consistent with prior studies, showing that SMP, a novel peptide-rich fraction derived from *S. marianum*, exerts hepatoprotective effects by enhancing antioxidative responses and suppressing proinflammatory cytokines such as TNF-α and IL-6 in CCl_4_-induced liver injury. SMP represents a distinct class of bioactive compounds derived from *S. marianum* and may possess pharmacological characteristics different from those of flavonolignan-based constituents. In our study, SMP markedly attenuated CCl_4_-induced liver injury by enhancing Nrf2-mediated antioxidant defenses, suppressing inflammatory responses, and modulating gut microbiota composition.

Macrophages represent the most abundant immune cell population in the liver and play a pivotal role in maintaining tissue and cellular homeostasis (Xu et al., 2024). In the context of acute liver injury, macrophage activation triggers the release of inflammatory mediators and chemokines (Liu Z. et al., 2022). The infiltration of macrophages into the liver parenchyma serves as both a hallmark and a contributing factor to hepatic inflammatory damage. Similarly, Ly6G + neutrophils exhibit excessive activation in acute liver injury, triggering an immune-mediated inflammatory response (Xie et al., 2025). Our research has demonstrated that SMP can reduce the number of F4/80+ macrophages and Ly6G + neutrophils in mice liver tissue, thereby decreasing the levels of inflammatory factors and chemokines and alleviating liver injury. SMP also reduced hepatic CD8^+^ T-cell infiltration. T lymphocyte subsets, specifically CD4^+^ and CD8^+^ T cells, are critically involved in the pathogenesis of liver diseases (Zhao et al., 2016). Nonetheless, investigations into the impact of CD4^+^ and CD8^+^ T cells on carbon tetrachloride (CCl_4_)-induced acute hepatotoxicity in murine models remain limited. Prior research has indicated that the depletion of CD8^+^ T cells confers protection against amodiaquine-induced hepatic injury in mice (Mak and Uetrecht, 2015). Furthermore, administration of human endometrial regenerative cells has been shown to markedly decrease the populations of both CD4^+^ and CD8^+^ T cells (Lu et al., 2016). These findings suggest that the reduction in hepatic CD8^+^ T-cell infiltration may contribute to the protective effects of SMP, although the underlying mechanisms remain to be elucidated. Future investigations will aim to elucidate the mechanisms by which SMP influences immune cells in the context of ALI prevention.

The TRAF3/NIK/NF-κB signaling pathway plays a pivotal role in regulating immune activation and inflammatory responses, both of which are central to the development and progression of liver injury (Tao et al., 2022). As reported by Liu et al. (2020), activation of the NF-κB pathway in liver cells leads to the upregulation of proinflammatory cytokines and contributes to liver damage. Consistent with these findings, we observed that CCl_4_ exposure activated the NIK/NF-κB axis, while SMP treatment effectively suppressed NIK and p52 expression and restored Nrf2 levels, thereby suggesting a potential inhibitory effect on NF-κB activation and attenuating hepatic inflammation. These results align with previous reports showing that silymarin reduces NF-κB activity (Zarpou et al., 2021). Our findings suggest that SMP may regulate this pathway at the level of NIK. In particular, SMP upregulated TRAF3 expression, which is involved in the regulation of NIK stability (Häcker et al., 2011). Mechanistically, TRAF3 facilitates the NIK ubiquitination and subsequent proteasomal degradation of NIK, a process that is essential for terminating sustained NF-κB signaling (Yu et al., 2020). Our co-immunoprecipitation and ubiquitination assays showed an interaction between TRAF3 and NIK, and demonstrated that SMP treatment significantly enhanced NIK ubiquitination. Additionally, cycloheximide-chase assays revealed that SMP shortened the half-life of NIK protein, providing further evidence for increased NIK degradation. This ubiquitin-mediated regulation is a critical post-translational mechanism by which the non-canonical NF-κB pathway is controlled. Under physiological conditions, NIK levels are tightly regulated by a TRAF2/TRAF3/cIAP1/2 complex, which continuously tags NIK for degradation via the proteasome (Maubach et al., 2019). Our findings therefore suggest that SMP may be associated with the regulation of NIK turnover, preventing its accumulation and blocking downstream inflammatory signaling. These findings are in line with previous work by Shiode et al. (2023) and Li Y. et al. (2020), who emphasized the importance of TRAF3-mediated NIK ubiquitination in maintaining hepatic immune homeostasis. Importantly, in Hepa1-6 cells overexpressing NIK, SMP not only reduced NF-κB transcriptional activity but also downregulated key inflammatory mediators such as TNF-α, CCL2, and CCL5, reinforcing its anti-inflammatory efficacy both *in vitro* and *in vivo*.

In recent years, the role of the gut microbiota in liver diseases, particularly alcoholic liver disease and NAFLD, has gained significant attention (Zafari et al., 2022; Khan et al., 2021). The gut‒liver axis serves as a key link between the gut microbiota and liver function, where microbial dysbiosis is often associated with chronic liver inflammation and fibrosis. Yao et al. (2022) reported that the gut microbiota modulates liver inflammation and immune responses through the production of microbial metabolites such as short-chain fatty acids, which reduce liver damage by promoting anti-inflammatory effects. Our results showed that SMP significantly remodeled the gut microbiota composition. This finding is consistent with previous studies that have demonstrated that silymarin improves liver function by modulating the gut microbiota in liver injury models (Shen et al., 2019; Jahanian et al., 2017; Li X. et al., 2020). The observed shift in microbiota composition suggests that SMP is associated with changes in the gut microbial community. However, whether these microbiota changes contribute to the hepatoprotective effects of SMP remains unclear. Interestingly, SMP induced substantial alterations in microbial community composition, suggesting a potential role of the gut–liver axis in mediating its hepatoprotective effects.

Our findings place SMP within a growing body of research on natural bioactive compounds with hepatoprotective effects. Like naringenin, which has been shown to alleviate NAFLD by downregulating the NF-κB pathway (Wang et al., 2020), and Maresin-1, which prevents liver fibrosis by targeting Nrf2 and NF-κB (Rodríguez et al., 2021), SMP also modulates the NF-κB pathway. However, the pharmacological properties of SMP, including its molecular selectivity and safety profile, require further investigation. In this study, we found that SMP not only targets key pathways such as the NF-κB pathway but also has a significant effect on the gut microbiota, a characteristic not fully explored in other hepatoprotective agents. This dual action-modulating intracellular signaling pathways and gut microbiota composition-suggests that SMP may exert hepatoprotective effects through multiple mechanisms, highlighting its potential for liver protection.

Despite these promising results, our study has several limitations. First, we focused primarily on a CCl_4_-induced ALI model, which mimics toxin-induced liver damage. While this model is useful for studying liver injury, chronic liver diseases, such as NAFLD and chronic hepatitis, present more complex and prolonged conditions. In addition, although three peptide sequences were identified in SMP, the active constituent(s) responsible for the observed effects remain undefined, and the complete peptide composition and detailed characterization of SMP remain to be determined. Future studies should investigate the efficacy of SMP in chronic liver disease models to evaluate its broader protective potential. Second, while we explored the gut microbiota and metabolomics in the context of SMP treatment, the precise mechanistic pathways by which SMP influences the microbiota and its metabolites remain unclear. The microbiota–metabolite correlation analysis was exploratory and was limited by the relatively small sample size (n = 5 per group); therefore, these associations do not establish causal relationships. The microbiome and metabolomics analyses were based on relatively small sample sizes, and causal experiments, such as microbiota depletion, transplantation, or targeted microbial/metabolite interventions, were not performed. Moreover, the metabolomics findings were based primarily on metabolite annotation rather than comprehensive confirmation using authentic standards and retention-time matching. Further research is needed to identify the specific microbial species and metabolites responsible for the liver-protective effects of SMP. Third, our study primarily evaluated the preventive effects of SMP, and whether SMP can effectively treat established ALI remains unclear. Therefore, the therapeutic efficacy of SMP after the onset of liver injury requires further investigation. Clinical studies are needed to further evaluate its safety and efficacy. Furthermore, only male mice were included in the present study, and potential sex-dependent effects of SMP were not evaluated. Pharmacokinetic and systemic exposure data were also not available, limiting our understanding of the absorption, distribution, metabolism, and exposure–response relationship of SMP.

## Conclusion

This study demonstrates that SMP exerts hepatoprotective effects in a preclinical CCl_4_-induced ALI model. SMP was associated with reduced NIK/NF-κB signaling activity, enhanced NIK ubiquitination and degradation in association with TRAF3, and alterations in gut microbiota composition. These findings suggest that modulation of the NIK/NF-κB pathway and gut microbiota may be associated with the hepatoprotective effects of SMP in this model. However, the precise molecular mechanism, including the specific ubiquitin linkage and the causal role of TRAF3, requires further investigation. Further studies are needed to characterize the active component(s) of SMP, define its pharmacokinetic and safety profiles, and determine its efficacy in established liver injury and other disease models before its therapeutic potential can be evaluated.

## Data Availability

The data presented in the study are deposited in the NCBI SRA repository, accession number PRJNA1394686.
