# Supplementary material for: Silybum marianum peptide-rich fraction alleviates acute liver injury by inhibiting TRAF3/NIK/NF-κB and is associated with changes in gut microbiota composition
Source: Front Pharmacol. 2026 Oct 5;17:1915943. doi: 10.3389/fphar.2026.1915943 (PMC13677544; doi:10.3389/fphar.2026.1915943)
Supplement: Supplementary file 1 [file DataSheet1.pdf]

Supplemental Material

Silybum marianum peptide alleviates acute liver injury by inhibiting TRAF3/NIK/NF-κB and regulating the gut microbiota

Ananda[1,#], Wenyan Han[2,#], Khurelbaatar Nyamdavaa[1], Xian Li[3,*], Tsend-Ayush Damba[1,*], Xiulan Su [3,*]

[1] International School of Mongolian Medicine, Mongolian National University of Medical Sciences. S. Zorig street-3, Ulaanbaatar 14210, Mongolia

[2] Clinical Laboratory, The Second Affiliated Hospital, Inner Mongolia Medical University, Hohhot 010091, Inner Mongolia Autonomous Region, P. R. China

[3] Clinical Medical Research Center, Inner Mongolia Bioactive Peptide Engineering Laboratory, The Affiliated Hospital of Inner Mongolia Medical University, Hohhot 010030, Inner Mongolia Autonomous Region, P. R. China

[#] Co-first authors

***Corresponding author 1: Xiulan Su,** Clinical Medical Research Center, Inner Mongolia Bioactive Peptide Engineering Laboratory, The Affiliated Hospital of Inner Mongolia Medical University, 1 Xinhua Street, Hohhot 010030, Inner Mongolia Autonomous Region, P. R. China

Email: Suxiulan11@163.com

Tel: +86-13904710692

***Corresponding author 2: Tsend-Ayush Damba,** International School of Mongolian Medicine, Mongolian National University of Medical Sciences. S. Zorig street-3, Ulaanbaatar 14210, Mongolia

Email: tsendayush@mnums.edu.mn

***Corresponding author 3: Xian Li，** Clinical Medical Research Center, Inner Mongolia Bioactive Peptide Engineering Laboratory, The Affiliated Hospital of Inner Mongolia Medical University, 1 Xinhua Street, Hohhot 010030, Inner Mongolia Autonomous Region, P. R. China

Email: li_xian1214@163.com

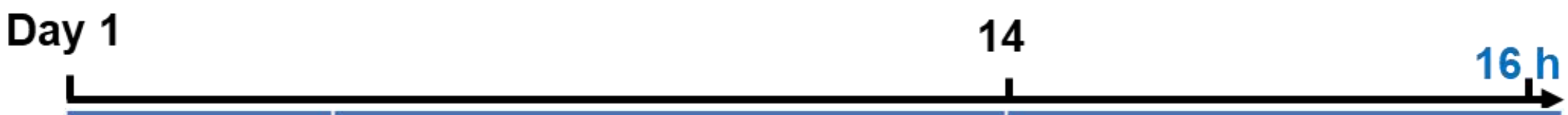

Group	Treatment (Day 1 – 14)	Treatment (Day 14)
Control	Saline, daily i.g.	Olive oil, i.p.
CCl_4	Saline, daily i.g.	10% CCl_4, 10 μL/g, i.p.
SMP-L	SMP 50 mg/kg, daily i.g.	
SMP-M	SMP 100 mg/kg, daily i.g.	
SMP-H	SMP 200 mg/kg, daily i.g.	

Supplementary Figure S1 Experimental timeline

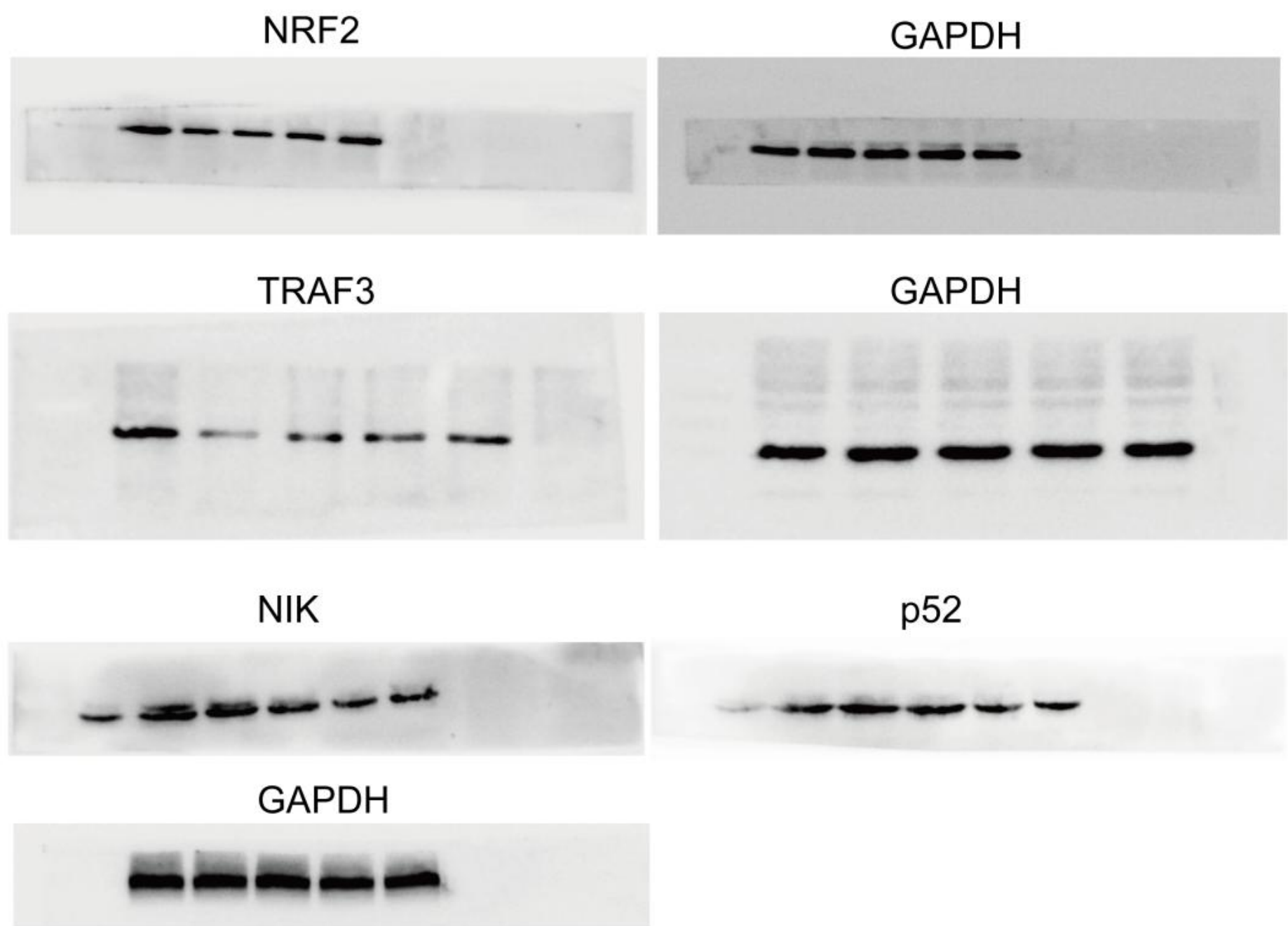

Supplementary Figure S2 Protein expression levels of NRF2, NIK, and p52 determined via western blot (the western blot lanes from left to right are: Control, CCl_4, CCl_4+SMP 50mg/kg, CCl_4+SMP 100mg/kg, CCl_4+SMP 200mg/kg, CCl_4+silymarin 100mg/kg).

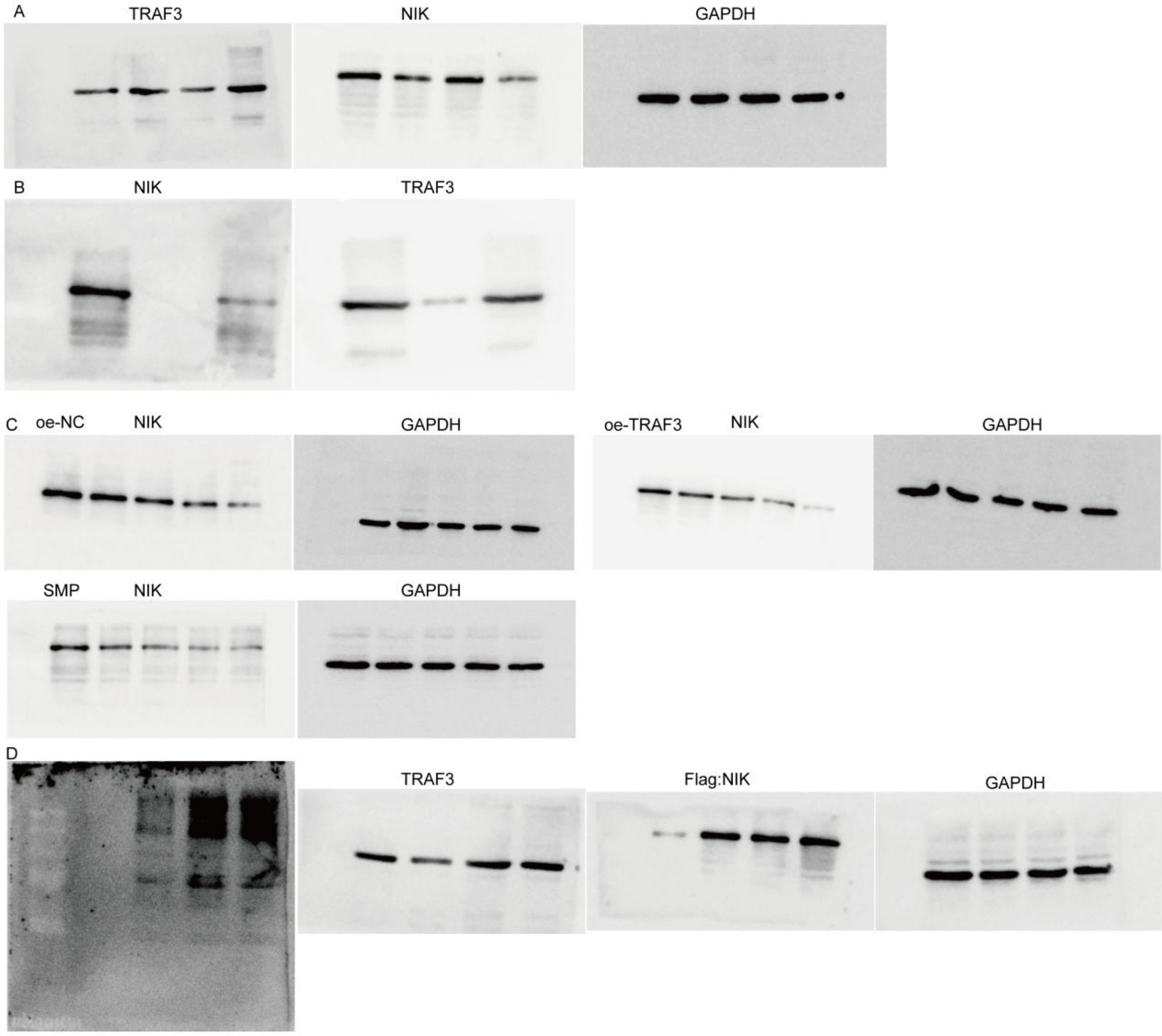

Supplementary Figure S3 (A) Protein expression levels of NIK and TRAF3 determined via western blot (the western blot lanes from left to right are: Control, SMP, oe-NC, oe-TRAF3); (B) The binding of the NIK and TRAF3 proteins was detected via a coimmunoprecipitation assay (the western blot lanes from left to right are: Input, lgG, TRAF3). (C) The half-life of the NIK protein was detected after different durations of CHX treatment (the western blot lanes from left to right are: 0h, 2h, 4h, 6h, 8h). (D) The level of NIK ubiquitination was analyzed after proteasome inhibitor (MG132) treatment (the western blot lanes from left to right are: SMP(-)TRAF3(-)Flag: NIK(-)HA-Ub(+)MG132(+), SMP(-)TRAF3(-)Flag:NIK(+)HA-Ub(+)MG132(+), SMP(-)TRAF3(+)Flag:NIK(-)HA-Ub(+)MG132(+), SMP(+)TRAF3(-)Flag:NIK(-)HA-Ub(+)MG132(+)).

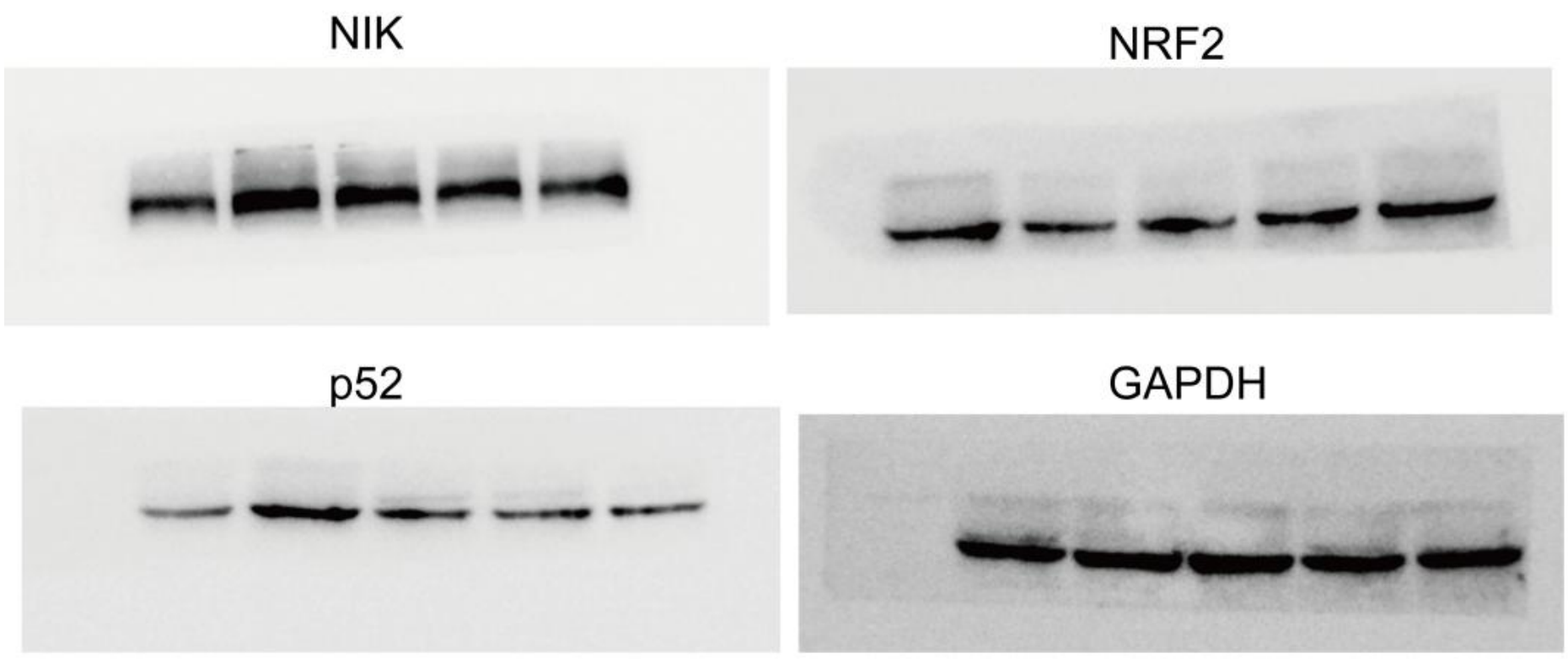

Supplementary Figure S4 Protein expression levels of NRF2, NIK, and p52 determined via western blot (the western blot lanes from left to right are: oe-vector, oe-NIK+solvent, oe-NIK+SMP 10μg/mL, oe-NIK+SMP 20μg/mL, oe-NIK+SMP 40μg/mL).
